# Machine Learning and Integrative Structural Dynamics Identify Potent ALK Inhibitors from Natural Compound Libraries

**DOI:** 10.3390/ph18081178

**Published:** 2025-08-10

**Authors:** Rana Alateeq

**Affiliations:** Department of Medical Laboratories, College of Applied Medical Sciences, Qassim University, Burydah 51452, Saudi Arabia; r.alateeq@qu.edu.sa

**Keywords:** ALK inhibitors, machine learning, molecular dynamics, virtual screening, binding free energy

## Abstract

**Background**: Anaplastic lymphoma kinase (ALK) is a validated oncogenic driver in non-small cell lung cancer and other malignancies, making it a clinically relevant target for small-molecule inhibition. **Methods**: Here, we report a computational discovery pipeline integrating structure-based virtual screening, machine learning-guided prioritization, molecular dynamics simulations, and binding free energy analysis to identify potential ALK inhibitors from a natural product-derived subset of the ZINC20 database. We trained and benchmarked eleven machine learning models, including tree-based, kernel-based, linear, and neural architectures, on curated bioactivity datasets of ALK inhibitors to capture nuanced structure-activity relationships and prioritize candidates beyond conventional docking metrics. **Results**: Six compounds were shortlisted based on binding affinity, solubility, bioavailability, and synthetic accessibility. Molecular dynamics simulations over 100 ns revealed stable ligand engagement, with limited conformational fluctuations and consistent retention of the protein’s structural integrity. Key catalytic residues, including GLU105, MET107, and ASP178, displayed minimal fluctuation, while hydrogen bonding and residue interaction analyses confirmed persistent engagement across all ligand-bound complexes. Binding free energy estimates identified ZINC3870414 and ZINC8214398 as top-performing candidates, with ΔG_total_ values of –46.02 and –46.18 kcal/mol, respectively. Principal component and dynamic network analyses indicated that these compounds restrict conformational sampling and reorganize residue communication pathways, consistent with functional inhibition. **Conclusions**: These results highlight ZINC3870414 and ZINC8214398 as promising scaffolds for further optimization and support the utility of integrating machine learning with dynamic and network-based metrics in early-stage kinase inhibitor discovery.

## 1. Introduction

### Background

Lung cancer is the leading cause of cancer-related mortality worldwide, with non-small cell lung cancer (NSCLC) accounting for the majority of cases [1]. Despite advances in diagnosis and treatment, outcomes for patients with advanced-stage NSCLC remain poor, in part due to tumor heterogeneity and resistance to targeted therapies [2]. Genomic profiling has identified several oncogenic drivers in NSCLC, among which rearrangements involving the anaplastic lymphoma kinase (ALK) gene represent a clinically actionable subset [3]. ALK encodes a receptor tyrosine kinase of the insulin receptor superfamily, with physiological roles in neural development during embryogenesis [4]. In NSCLC, chromosomal rearrangements most frequently involving the EML4 gene lead to the expression of constitutively active fusion proteins such as EML4-ALK [5]. These fusions drive tumor progression through activation of downstream signaling pathways including PI3K/AKT, RAS/ERK, and JAK/STAT, supporting uncontrolled proliferation and survival [6]. Targeted inhibition of ALK has transformed the therapeutic landscape for ALK-positive NSCLC. Multiple ALK tyrosine kinase inhibitors (TKIs), including crizotinib, ceritinib, alectinib, brigatinib, and lorlatinib, have demonstrated clinical efficacy, improving progression-free survival and overall response rates [7,8]. Although multiple generations of ALK tyrosine kinase inhibitors have been developed, resistance remains a major barrier to durable clinical responses [9]. Many of these inhibitors share conserved core scaffolds, limiting their activity against resistant ALK variants [9,10]. As new resistance mutations continue to emerge, the effectiveness of existing therapies diminishes, underscoring the need for structurally distinct compounds [11]. However, therapeutic benefit is often limited by the development of resistance [12]. This may arise through secondary mutations in the kinase domain, activation of bypass signaling pathways, or suboptimal drug penetration into the central nervous system. [13]. For example, gatekeeper mutations such as L1196M reduce binding of first-generation TKIs like crizotinib, while compound mutations may confer resistance to even third-generation drugs like lorlatinib [14]. These challenges highlight the need for alternative chemical scaffolds capable of targeting resistant ALK variants [15]. Genomic profiling of lung adenocarcinoma cohorts has identified ALK gene rearrangements in approximately 3–7% of NSCLC cases, predominantly in younger, non-smoking patients [16,17]. These alterations most commonly involve EML4-ALK fusions, which result in constitutive activation of ALK activity and are considered early oncogenic events [17]. Analysis of The Cancer Genome Atlas (TCGA) LUAD dataset via cBioPortal confirms that ALK fusions are largely mutually exclusive with EGFR, KRAS, and ROS1 alterations, underscoring ALK’s role as a distinct and actionable driver in a molecularly defined NSCLC subset [18].

Natural product-inspired compounds represent a promising source of new therapeutics due to their structural complexity and evolutionary optimization for biological activity [19]. Their high stereochemical complexity, diversity of functional groups, and structural novelty often allow interactions with targets considered undruggable by synthetic compounds [20]. However, experimental screening of large natural product libraries is time- and resource-intensive, making computational approaches essential for early-stage prioritization [21]. Computational screening of chemically diverse natural product-like libraries provides a rational strategy for identifying candidates with unique binding modes and potential to overcome resistance-associated limitations of existing ALK inhibitors [22]. Furthermore, drug discovery efforts have largely focused on synthetic libraries, which may not fully reflect the chemical diversity and biological relevance of natural product scaffolds [23]. Natural product-like compounds offer a promising alternative due to their structural complexity and resemblance to biologically active molecules [24]. Compounds curated in the ZINC20 database represent a chemically diverse and readily accessible subset of natural product-like molecules suitable for virtual screening [25]. These molecules frequently exhibit favorable drug-like properties, including absorption, distribution, metabolism, excretion, and toxicity (ADMET) profiles. In this study, we conducted a structure-based virtual screening of the ZINC20 natural product-like subset to identify potential inhibitors of the ALK domain. Targeting the PHA-E429 binding site, we used molecular docking to prioritize ligands based on binding affinity and interaction specificity [26]. Following docking, top candidates were evaluated using molecular dynamics simulations to assess binding stability and conformational persistence within the ALK active site. Binding free energy calculations were carried out using the MM/GBSA method to estimate the thermodynamic favorability of interaction. In addition, predicted pharmacokinetic and toxicity parameters were assessed to support compound drug-likeness.

## 2. Results

### 2.1. Co-Crystal Structure of ALK with PHA-E429

**Figure 1:**
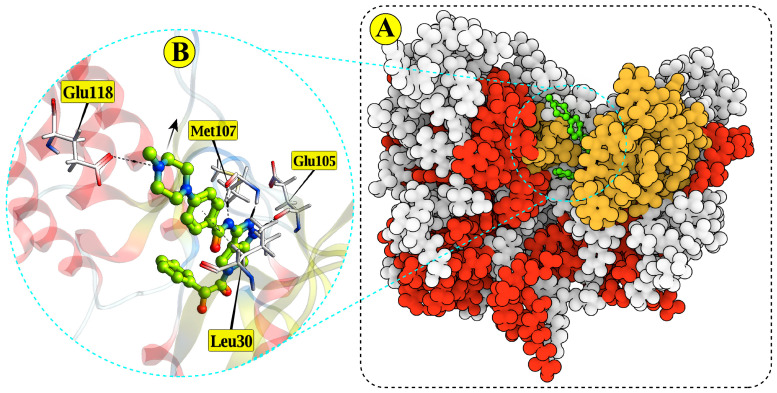
(**A**) Surface representation of the ALK (anaplastic lymphoma kinase) kinase domain showing the co-crystallized inhibitor PHA-E429 (green) bound within the ATP-binding pocket (yellow). The protein surface is colored according to secondary structure elements: α-helices in red, β-sheets in yellow, and coils in white, providing structural context for the binding interface. (**B**) Close-up view of the binding pocket highlighting key interactions between PHA-E429 and residues Glu105, Glu118, Met107, and Leu30.

The crystal structure of human anaplastic lymphoma kinase (ALK) bound to the inhibitor PHA-E429 (PDB ID: 2XBA) was used to define the active site. The structure, resolved at 1.95 Å, shows the ligand positioned deep within the catalytic cleft (Figure 1). PHA-E429 has a reported IC_50_ of 0.014 ± 0.064 μM, indicating high potency. This complex was used to validate the docking protocol and define key active site residues. Interaction analysis revealed multiple stabilizing contacts, including hydrogen bonds with Glu118 (3.6 Å, −1.2 kcal/mol), Met107 (2.97 Å and 2.72 Å, −2.4 and −4.3 kcal/mol), and Glu105 (2.65 Å, −3.6 kcal/mol). Ionic interactions with Glu118 (3.6 Å, −1.5 kcal/mol) and a π–H contact with Leu30 (4.5 Å, −0.8 kcal/mol) further supported the ligand’s binding stability.

### 2.2. Dataset and Compound Distribution

**Figure 2:**
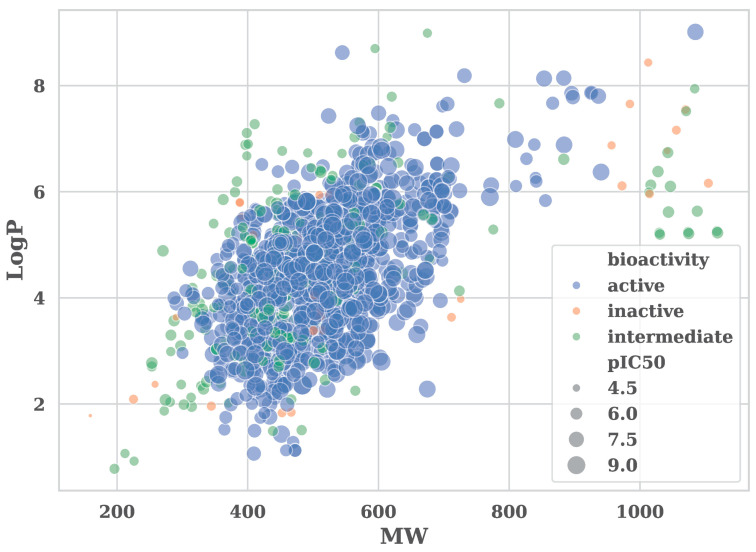
Distribution of active (blue), inactive (orange), and intermediate (green) compounds based on two molecular descriptors (molecular weight and LogP). Marker size is scaled by pIC_50_.

Bioactivity data for human ALK was retrieved from the ChEMBL database (ChEMBL ID: CHEMBL279). After filtering for valid IC_50_ values and SMILES strings, a total of 1259 compounds were retained for model development. IC_50_ values were transformed to pIC_50_, and compounds were categorized as active (pIC_50_ ≥ 6), inactive (pIC_50_ < 5), or intermediate (5 ≤ pIC_50_ < 6). Only active and inactive compounds were used for classification. The distribution of compounds by molecular weight (MW) and lipophilicity (LogP) revealed that most active compounds clustered between 400 and 600 Da and LogP values of 3–5. Intermediate and inactive compounds were more dispersed. A 2D distribution plot based on molecular weight and LogP showed partial overlap among activity classes, consistent with the chemical diversity of the dataset (Figure 2).

Physicochemical properties of the compounds were analyzed across activity classes to assess trends associated with ALK inhibition. Active compounds showed lower molecular weight and fewer hydrogen bond donors compared to inactive compounds. Differences in LogP and hydrogen bond acceptors were less pronounced. These distributions suggest that moderate lipophilicity and reduced polarity may favor ALK activity in this dataset (Figure S1).

### 2.3. AI Model Performance and Selection

**Figure 3:**
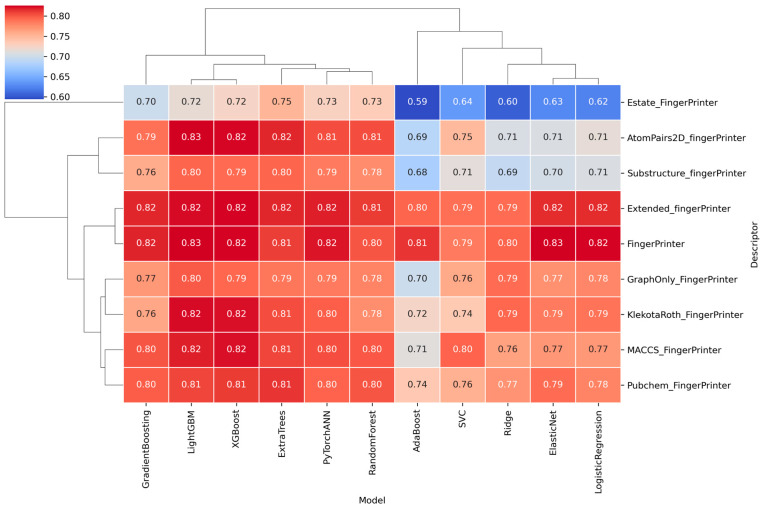
Clustering heatmap of AUC scores across top models and fingerprints. Average AUC values for each model-descriptor combination are displayed as a clustered heatmap.

Eleven supervised learning algorithms were evaluated across nine molecular fingerprint types to classify ALK inhibitors. Models included ensemble methods (Random Forest, XGBoost, LightGBM, AdaBoost, ExtraTrees), linear classifiers (Logistic Regression, Ridge, ElasticNet), a support vector machine (SVC), and an artificial neural network (PyTorchANN). Models were evaluated using repeated random 80:20 train–test splits over 100 iterations to ensure robust performance estimation. The average AUC scores across top combinations are summarized in a hierarchical clustering heatmap (Figure 3).

**Figure 4:**
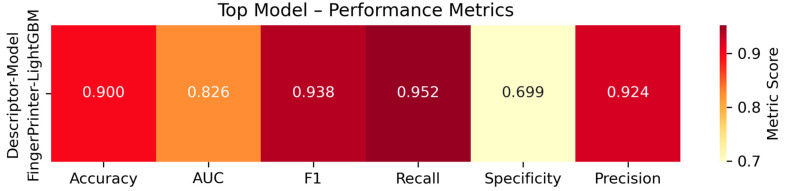
Performance metrics of the top-ranked classification model. Validation performance of the LightGBM classifier trained on the CDKextended fingerprint. Metrics include accuracy (0.900), AUC (0.826), F1 score (0.938), recall (0.952), specificity (0.699), and precision (0.924).

Performance varied with descriptor type, with fingerprints such as CDK, CDKextended, Klekota-Roth, and MACCS showing consistently higher predictive performance across models. Among classifiers, ensemble methods outperformed linear models, and PyTorchANN achieved comparable results. LightGBM trained on CDKextended fingerprints achieved the highest validation performance (Figure 4), with an accuracy of 0.900, AUC of 0.826, F1 score of 0.938, recall of 0.952, and precision of 0.924. Specificity was relatively lower (0.699), suggesting a conservative bias in predicting inactive compounds. Detailed comparison of Random Forest, XGBoost, and PyTorchANN across all fingerprints is shown in Figure S2. ROC curves for all model–descriptor pairs are provided in Figures S3–S11. Corresponding confusion matrices are included in Figures S12–S21. The top model (LightGBM + CDKextended fingerprint) was selected for downstream virtual screening based on its balanced performance and generalization. The best-performing models and descriptor combinations, ranked by the average of AUC and Accuracy (AUC_Acc_Avg), are summarized in Table S1.

### 2.4. Molecular Docking Assay

Ligand-based screening was performed on the ZINC20 natural product subset using the top-performing AI model (LightGBM with CDKextended fingerprint), yielding 50 high-confidence candidates. These compounds were docked into the ALK active site defined by the co-crystallized ligand PHA-E429. Docking scores ranged from −6.48 to −10.32 kcal/mol, with RMSD values between 1.04 and 3.71 Å, indicating stable predicted poses. All compounds were further assessed for physicochemical and pharmacokinetic properties, including drug-likeness and toxicity predictions. Based on docking score, pose quality (RMSD), and pharmacokinetic profiles, six compounds were selected as top candidates (Table 1). Among them, ZINC3870414 exhibited the strongest predicted binding affinity (−10.31 kcal/mol) with an RMSD of 1.8 Å, outperforming the reference inhibitor PHA-E429 (−8.78 kcal/mol, RMSD 1.2 Å). The remaining five compounds also showed favorable binding, with scores between −9.46 and −8.90 kcal/mol and RMSD values from 1.2 to 2.7 Å. These results support their potential as lead scaffolds for further development.

**Figure 5:**
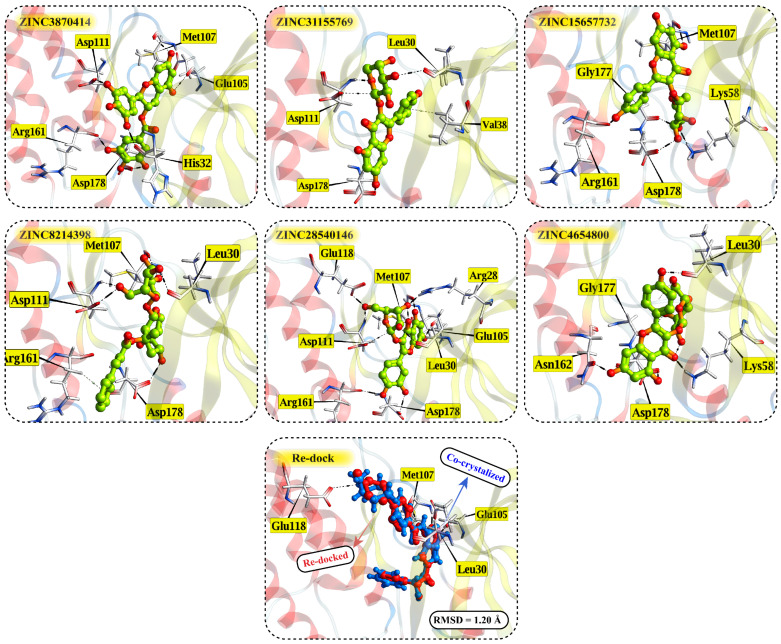
Binding poses of top-ranked ZINC20 natural product-like compounds in the ALK active site. Each panel shows the predicted binding mode of one selected compound (green) with key interacting residues labeled. Hydrogen bond and ionic contacts are shown for ZINC3870414, ZINC31155769, ZINC15657732, ZINC8214398, ZINC28540146, and ZINC4654800. The bottom panel displays the redocking validation of the co-crystallized inhibitor PHA-E429, with the redocked pose (red) overlaid on the original crystal structure (blue). The resulting RMSD of 1.20 Å confirms the reliability of the docking protocol.

Interaction analysis of the six selected ZINC20-derived compounds revealed multiple stable contacts with key residues in the ALK binding site, consistent with the binding pose of the co-crystallized ligand PHA-E429 (Figure 5). The highest-ranked compound, ZINC3870414, formed seven hydrogen bonds with Glu105, Met107, His32, Asp111, Arg161, and Asp178, with interaction distances ranging from 2.54 to 3.55 Å and energies between −0.6 and −3.6 kcal/mol. Notably, interactions with Glu105, Met107, and Asp178 reflected those observed in the control compound, supporting a conserved binding mode. ZINC31155769 also showed a strong profile, forming six interactions including a π–H contact with Val38 and hydrogen bonds with Asp111, Leu30, and Asp178. These residues overlap with those engaged by PHA-E429, suggesting that ZINC31155769 occupies a similar region within the active site. ZINC15657732 formed five hydrogen bonds, notably with Met107, Arg161, Asp178, and Gly177, along with a strong hydrogen bond acceptor interaction with Lys58 (−6.0 kcal/mol). This Lys58 contact, not observed in the control, may contribute to its binding stability and could represent a differentiating feature. ZINC8214398 exhibited multiple favorable contacts including hydrogen bonds with Asp111, Leu30, Met107, and Asp178, in addition to π–H stacking with Arg161. These contacts were mostly aligned with the binding profile of PHA-E429. ZINC28540146 showed extensive interaction with Glu105, Met107, Glu118, Leu30, Asp111, Asp178, Arg161, and Arg28, including both donor and acceptor interactions. This interaction pattern, with nine hydrogen bonds and a π–H contact, most closely resembled the interaction complexity of the control compound and supports its potential as a strong binder. ZINC4654800 displayed hydrogen bonds with Asn162, Asp178, Gly177, and Leu30, and a strong acceptor interaction with Lys58 (−11.7 kcal/mol), the most energetically favorable contact among all tested compounds. These results suggest a uniquely stabilized binding mode distinct from PHA-E429. Together, these interaction profiles support the structural stability and site-specific binding of all six selected compounds. Many engaged the same residues as the control ligand, while others revealed unique contacts that may enhance affinity or specificity. Detailed interaction data are provided in Supplementary Table S2.

### 2.5. Drug-like Properties Evaluation of the Selected Hits

The six final compounds were evaluated for key physicochemical properties relevant to oral bioavailability and drug-likeness using the SwissADME platform. All compounds had molecular weights ranging from 418.35 to 494.49 Da and complied with the recommended thresholds for hydrogen bond acceptors (≤10–11) and donors (≤8). The number of rotatable bonds per compound ranged from 3 to 7, consistent with good conformational flexibility without compromising binding specificity. Log Po/w values (consensus lipophilicity) varied from −0.29 to 0.99, reflecting moderate lipophilicity, while water solubility predictions (Log S, ESOL) ranged from −2.19 to −3.56. All compounds were predicted to be water-soluble. These profiles indicate that the shortlisted hits possess favorable molecular and physicochemical characteristics consistent with drug-likeness and bioavailability. Full data are presented in Supplementary Table S3. Absorption and distribution parameters of the six selected compounds were assessed using the SwissADME server. Synthetic accessibility scores ranged from 4.20 to 6.13, indicating moderate ease of synthesis. None of the compounds were predicted to cross the blood–brain barrier (BBB), a desirable trait for avoiding central nervous system-related side effects. All compounds, except ZINC15657732, were predicted not to be substrates of P-glycoprotein (P-gp), reducing the likelihood of efflux-mediated clearance. Bioavailability scores ranged from 0.55 to 0.56 across the series, suggesting consistent oral absorption potential. These results support the suitability of the selected compounds for further development as kinase inhibitors with minimal CNS exposure. Detailed parameters are available in Supplementary Table S4.

### 2.6. Dynamic Stability of the Protein–Ligand Complexes

RMSD analysis was performed over 100 ns simulations to assess the structural stability of ALK in complex with the selected ligands. The apo form of ALK showed gradual deviations, stabilizing around 2.5 Å after ~60 ns, indicating intrinsic flexibility in the absence of a bound ligand. In contrast, the control complex with PHA-E429 (2XBA) stabilized more rapidly, reaching equilibrium near 2.2 Å by ~30 ns. All ligand-bound systems demonstrated initial RMSD fluctuations during the first 20–30 ns, followed by stabilization indicative of stable complex formation. ZINC3870414 maintained a stable trajectory with RMSD fluctuations around 2.0–2.3 Å after 30 ns, similar to the reference compound. ZINC31155769 and ZINC15657732 stabilized around 2.5–2.7 Å, showing moderate flexibility but consistent structural integrity. ZINC8214398 and ZINC28540146 exhibited slightly higher deviations (~2.8–3.0 Å), with stability achieved after ~40 ns. Notably, ZINC4654800 formed a stable complex early, reaching a plateau near 2.1 Å and maintaining consistent conformational behavior throughout the simulation. These RMSD profiles suggest that all six ligands form dynamically stable complexes with ALK, with fluctuation magnitudes comparable to or better than the apo and control systems (Figure S22).

### 2.7. Residue Flexibility and Binding Site Stability

RMSF analysis was conducted to assess residue-level flexibility across the simulation trajectories. The apo structure displayed prominent fluctuations in several loop and terminal regions, particularly residues 30–55, 115–125, and 250–270, with RMSF values exceeding 3.5 Å. These fluctuations were reduced upon ligand binding, indicating dampened mobility and increased local stabilization. For the reference inhibitor (2XBA), reduced flexibility was observed at key binding site residues including Glu105, Met107, and Glu118, consistent with tight engagement seen in the crystal structure. All six ligand-bound complexes demonstrated similar suppression of fluctuations in these residues. ZINC3870414, the top-ranking compound, showed minimal mobility in active site regions, particularly around Glu105, Met107, Leu30, and Asp111, suggesting tight and stable binding. ZINC31155769 and ZINC15657732 also showed localized stabilization at the active site but retained moderate flexibility in distal loop regions. ZINC28540146 and ZINC4654800 exhibited broader suppression of fluctuations across the protein, particularly in residues 100–150 and 160–180, encompassing known binding and allosteric regions. ZINC8214398 showed a slightly more dynamic profile, though active site residues remained stable. Overall, the RMSF data confirm that ligand binding reduces the conformational mobility of ALK, particularly within the active site, supporting the formation of stable and specific complexes (Figure S23).

### 2.8. Compactness of the Protein Structure

To assess the global compactness of the protein during simulations, the Rg was monitored over 100 ns. The apo ALK structure showed a gradual decrease in Rg from ~20.6 Å to ~20.2 Å, indicating slight compaction in the absence of a ligand. In contrast, the ALK–PHA-E429 complex (2XBA) maintained a relatively stable Rg around 20.4–20.5 Å, reflecting a preserved structural core upon ligand binding. All ligand-bound complexes displayed minimal fluctuation in Rg, suggesting that none of the compounds induced significant destabilization or unfolding. ZINC3870414 and ZINC28540146 exhibited a slight decrease in Rg over time, reaching ~20.2 Å, which is consistent with increased compactness and potential stabilization of the protein fold. ZINC31155769, ZINC15657732, ZINC8214398, and ZINC4654800 maintained stable Rg values within the 20.4–20.6 Å range, similar to the control. These results confirm that the binding of all selected ZINC20 compounds preserves or modestly enhances the compactness of ALK, supporting their compatibility with the native protein structure (Figure S24).

### 2.9. Protein Dominant Collective Motions

**Figure 6:**
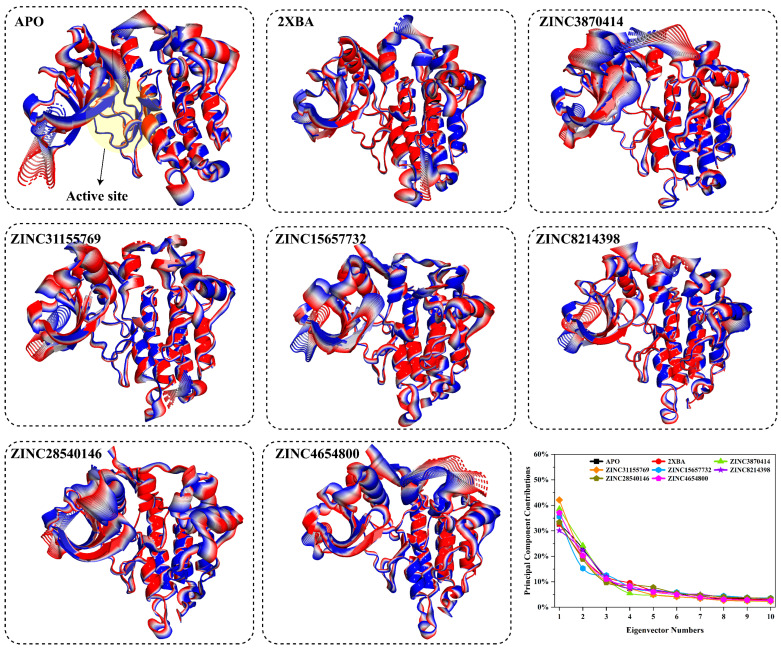
Principal component analysis of ALK in APO form and in complex with selected ligands. Structures represent conformational transitions along the first principal component (PC1) over 100 ns, with the start (0 ns) in blue and the end (100 ns) in red. Greater separation between blue and red regions indicates more extensive motion. The graph (bottom right) shows the percentage contribution of 10 PCs to the total motion for each system.

PCA was performed to characterize the dominant collective motions of ALK during molecular dynamics simulations. The first principal component (PC1), representing the most significant mode of motion, was visualized through structural transitions over time, with blue indicating the initial conformation (0 ns) and red the final frame (100 ns). These motions provide insight into large-scale domain shifts and conformational rearrangements. In the apo state, ALK exhibited a high degree of motion with a PC1 contribution of 32.66%. This flexibility was reduced upon ligand binding in several complexes. Notably, the PHA-E429-bound form (2XBA) showed a comparable contribution (32.70%), indicating moderate suppression of global movements. ZINC8214398 displayed the lowest PC1 contribution (30.14%), suggesting maximal dampening of dominant motions among all ligand-bound systems. Conversely, ZINC31155769 induced the highest PC1 variance (42.14%), followed by ZINC3870414 (38.59%) and ZINC4654800 (37.06%), reflecting more extensive structural shifts over the simulation. ZINC28540146 and ZINC15657732 exhibited intermediate contributions of 33.33% and 35.58%, respectively. Cartoon representations of each system demonstrate the time-evolved displacement of protein regions from blue (start) to red (end), highlighting the extent and location of dominant movements. These results suggest that while some ligands, such as ZINC8214398, ZINC28540146, and 2XBA, rigidify the protein core and limit conformational mobility, others like ZINC31155769, ZINC3870414, and ZINC4654800 permit and induce broader conformational exploration. These differences may reflect varying degrees of dynamic modulation upon ligand binding, which could influence downstream signaling and functional outcomes (Figure 6).

**Figure 7:**
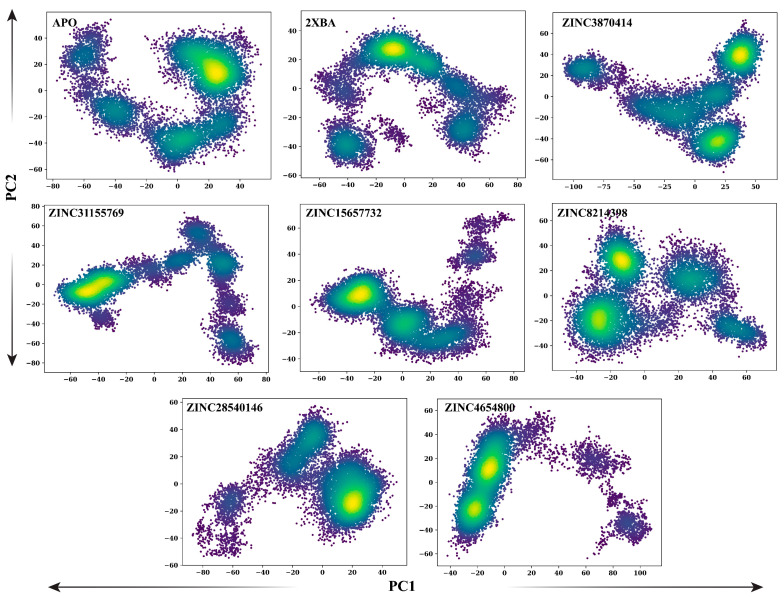
Two-dimensional PCA projections (PC1 vs. PC2) showing conformational state distributions of ALK in apo form, control (2XBA), and in complex with selected ZINC20 ligands over 100 ns simulations. Each dot represents a frame along the simulation trajectory, with yellow regions denoting high-density (low energy) conformations.

To explore the diversity and stability of conformational states sampled during simulation, projections along the first two principal components (PC1 vs. PC2) were generated for each system. These plots represent the free energy landscape, with dense yellow regions indicating more frequently sampled and energetically stable conformations. The apo form of ALK displayed a broad conformational space with at least three distinct low-energy basins, reflecting high structural flexibility and dynamic transitions. Binding of the reference inhibitor PHA-E429 (2XBA) reduced this heterogeneity, yielding two dominant stable states with more restricted motion. Ligand-bound complexes exhibited varied degrees of conformational clustering. ZINC3870414 and ZINC31155769 displayed compact landscapes with one or two high-density states, consistent with relatively stable binding and restrained dynamics. ZINC15657732, ZINC28540146, and ZINC4654800 also demonstrated limited conformational spread, each sampling two to three energetically preferred states. In contrast, ZINC8214398 resembled the apo form more closely, with a broader distribution and multiple moderate-density basins, suggesting higher residual flexibility. Overall, the PCA projections confirm that most ligand-bound systems adopt fewer and more defined conformational states compared to the apo protein, supporting ligand-induced stabilization of the protein structure. ZINC3870414, ZINC31155769, and ZINC4654800 showed highly confined energy landscapes, suggesting strong conformational restriction. ZINC15657732 and ZINC28540146 exhibited two to three dominant basins, reflecting moderate structural stability. In contrast, ZINC8214398 displayed a broader conformational spread with multiple basins, similar to the apo form, indicating a relatively flexible dynamic profile (Figure 7).

### 2.10. Hydrogen Bond Interaction Analysis

To evaluate the stability and specificity of ligand interaction, hydrogen bond interactions between ALK and the selected compounds were tracked across the simulation trajectories (Figures S25–S27). In the reference complex with PHA-E429, GLU105 (73.05%) and MET107 (60.77%) displayed sustained hydrogen bonding, highlighting their central role in ligand attachment. ASP178 also contributed through moderate interactions (19.68%, 18.63%). Among the screened ligands, ZINC3870414 formed stable hydrogen bonds with GLU105 (84.99%), ASP178 (57.90%, 55.21%, 38.65%, 30.43%), GLU75 (53.15%), ALA34 (24.00%), and GLY36 (23.97%). Additional residues PHE179, HIE32, GLY177, MET107, GLY33, ASP111, ARG28, LYS58, and ARG161 were involved at lower frequencies (bond life < 10%). ZINC31155769 maintained strong interactions with GLU105 (98.90%), GLU75 (86.20%, 10.75%), ASP178 (34.98%, 15.05%), MET107 (33.83%), and HIE32 (21.86%, 19.01%). Supporting residues included ARG161, LEU30, ASP111, ALA34, GLY31, PHE179, and LYS58 (bond life <10%). ZINC15657732 interact consistently with GLU105 (57.36%), ASP178 (41.76%, 23.48%), GLY177 (19.86%), MET107 (15.25%, 12.36%), ARG161 (13.54%), and GLU40 (10.95%). Supporting contributions came from ASP111, ALA34, GLU75, LYS58, LEU30, GLY36, HIE32, and ARG28 (bond life < 10%). ZINC8214398 demonstrated persistent bonding with ASP111 (37.10%), GLU105 (33.59%), GLY177 (28.44%, 23.16%), and LEU106 (5.49%), supported by interactions with MET107, ASP178, ARG161, LYS58, and HIE32 (bond life < 10%). ZINC28540146 interacted strongly with GLU105 (97.40%) and formed additional hydrogen bonds with ASP178 (18.79%, 14.55%), LEU30 (15.37%), ASP111 (13.91%, 12.37%), and HIE32 (10.95%). Residues such as GLY177, GLY36, GLY33, ARG161, and LYS58 were also involved (bond life <10%). ZINC4654800 established stable interactions with GLU105 (85.70%), GLU75 (36.59%, 24.37%, 23.65%, 17.00%, 11.42%), LYS58 (12.88%, 12.75%, 11.66%), GLY177 (11.97%), and ASP178 (11.35%, 11.23%). Additional hydrogen bonds involved ARG161, LEU30, ASP111, ASN162, MET107, ALA34, and GLY36 (bond life < 10%). Across all complexes, GLU105 emerged as a conserved hydrogen bonding site, consistent with its anchoring function in the reference complex. ASP178, MET107, and GLY177 were also frequently engaged, reflecting a recurrent interaction motif associated with ligand binding stability. Complete interaction details are provided in Table S5.

### 2.11. Dynamic Residue Network and Shortest Path Analysis

**Figure 8:**
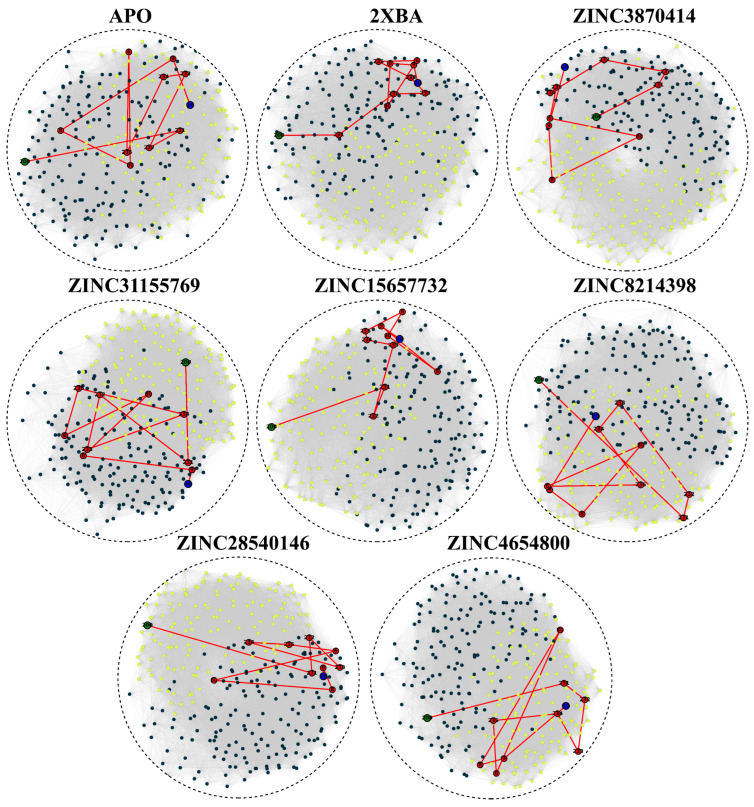
Dynamic network analysis reveals altered communication paths in apo and ligand-bound ALK complexes. The shortest communication pathways between key regions are mapped for the apo structure and each ligand-bound form. Different colors indicate distinct residue communities identified in the dynamic network analysis.

To investigate how ligand binding influences long-range residue communication within ALK, we analyzed the shortest communication paths across the protein network (Figure 8). The apo form exhibited a path that traversed THR10, SER11, and SER12 in the N-terminal region, extended through LEU77 and a long loop involving LYS260 to GLY254, and concluded via the C-terminal segment including PHE249, GLU248, LEU247, VAL201, and PRO200. This path suggests a broad and diffuse communication network in the absence of a ligand. In the reference 2XBA complex, the path was redirected through residues in the mid-domain, GLY234, TYR235, and MET236, followed by SER240 to GLN244, and finally reaching PRO200. This shift indicates a ligand-induced compaction of the communication route toward a more localized segment of the protein. ZINC3870414 established a unique path incorporating early binding pocket residues including CYS5 and THR10, followed by ARG120, PRO121, and loop region residues such as PRO126 and SER128. The path extended through PRO170, GLY171, VAL173 and terminated via LEU199 and PRO200. The inclusion of several proline residues suggests constrained but stable communication flow. ZINC31155769 preserved a path similar to the apo state, including THR10, SER11, and residues from PRO259 through GLY254, before converging on the C-terminal region via PHE249 to PRO200. This indicates partial retention of the native communication pattern. ZINC15657732 and ZINC28540146 displayed near-identical paths to 2XBA, favoring residues GLY234 to GLN244 and terminating at PRO200. Their alignment with the control path reflects similar allosteric effects. ZINC8214398 introduced a mixed path, starting with CYS5 and THR10, progressing through ARG120 to PRO126 and SER127, then through ILE185 and TYR186 before converging via PRO170, ARG172, and ending at LEU199 and PRO200. This pathway suggests influence on both core and extended regions. ZINC4654800 utilized a distinct route incorporating more peripheral residues including ILE307, PRO306, LEU305, and ALA304, and traversed central sites like ALA130, ARG183, and ILE185. The path concluded through ALA174, VAL173, LEU199, and PRO200, indicating that this ligand influenced distal and flexible segments of the protein. Collectively, all ligand-bound systems exhibited shorter, more localized communication routes compared to the apo protein. Many paths consistently included THR10, LEU199, and PRO200, underscoring their roles in intramolecular signaling and structural integration. Notably, control and most effective ligands (ZINC3870414, ZINC15657732, and ZINC28540146) favored mid-domain and terminal convergence, suggesting stabilization of critical communication routes.

### 2.12. Binding Free Energy Calculations

Binding free energy calculations were performed using the MM-GBSA approach to evaluate the thermodynamic stability of the ligand–ALK complexes. Among all, ZINC3870414 showed the most favorable total binding free energy (ΔG_TOTAL_ = −46.02 ± 0.12 kcal/mol), surpassing the reference inhibitor PHA-E429 (ΔG_TOTAL_ = −36.57 ± 0.12 kcal/mol). ZINC8214398 also demonstrated strong binding affinity (ΔG_TOTAL_ = −46.18 ± 0.12 kcal/mol), followed by ZINC15657732 (−40.89 ± 0.16 kcal/mol), ZINC4654800 (−40.56 ± 0.23 kcal/mol), and ZINC28540146 (−39.86 ± 0.17 kcal/mol). ZINC31155769 displayed slightly lower but still favorable binding energy (−39.69 ± 0.36 kcal/mol). The van der Waals (ΔE_VDW_) and electrostatic (ΔE_EL_) contributions were major driving forces across all complexes. ZINC3870414 and ZINC15657732 benefited from strong van der Waals interactions, while electrostatic components were particularly pronounced in ZINC3870414 and ZINC4654800. The solvation energies (ΔG_SOLV_) varied but were less dominant in determining binding strength. Overall, the MM-GBSA results indicate that the top-scoring compounds, especially ZINC3870414 and ZINC8214398, bind more stable than the control, supporting their potential as effective ALK inhibitors. The detailed binding energy components for each complex are presented in Table 2.

## 3. Discussion

Anaplastic lymphoma kinase (ALK) has been implicated as a key driver in several malignancies, including non-small cell lung cancer and anaplastic large cell lymphoma, through various activating mutations, fusions, and amplifications [27,28]. Its role in sustaining proliferative signaling and cell survival has established ALK as a validated oncogenic target, leading to the development of several ATP-competitive inhibitors [29]. However, the emergence of resistance mutations and limited efficacy in some patient subsets continue to limit the clinical durability of these therapies, necessitating the discovery of alternative small molecules with improved binding characteristics and broader inhibitory profiles [9,30]. Structure-guided drug discovery remains a vital strategy for identifying novel ALK inhibitors, particularly those that can engage resistant variants or modulate conformational plasticity through allosteric or cryptic binding sites [31]. In this work, we applied a multi-step in silico pipeline that integrates AI-driven model training with structure-based screening, molecular docking, dynamics simulations, and free energy calculations to identify candidate ALK inhibitors [26], Our goal was to benchmark these compounds based on predicted binding strength, interaction persistence, and conformational stability throughout the simulation process.

Our initial virtual screening of the ZINC20 database, guided by the crystallographic binding pose of PHA-E429 (PDB ID: 2XBA), prioritized compounds based on docking score and binding pose congruence. Top candidates consistently aligned with key catalytic residues in the ATP-binding site, including GLU105 and MET107, which are known to be critical for ALK inhibition and conserved across kinase families [32,33]. To refine candidate selection beyond docking metrics, we trained eleven supervised machine learning models using a curated dataset of known ALK inhibitors and inactives from ChEMBL. These models used molecular fingerprints and selected physicochemical descriptors as input features. The best-performing models were applied to rescore and refine the top-ranked candidates, integrating predictive features beyond docking metrics. From this two-stage filtering approach, six ZINC compounds were selected based on consensus ranking and ADME properties. According to SwissADME predictions, they displayed acceptable molecular weight, hydrogen bond features, rotatable bonds, and lipophilicity. Notably, most compounds were predicted non-substrates of P-glycoprotein, indicating good intestinal absorption and reduced efflux potential [34]. Additionally, synthetic accessibility scores ranged from 4.2 to 6.1, indicating realistic prospects for chemical synthesis and optimization. The overall ADME profile particularly for ZINC3870414 and ZINC31155769 was consistent with drug-likeness thresholds and justified their progression to molecular dynamics simulations. These predictions also corroborated previous studies that underscore the value of integrating ADME filters early in virtual screening workflows to reduce attrition rates in downstream experimental validation [35].

Molecular dynamics simulations were employed to refine our understanding of ligand-induced conformational stability and interaction dynamics. RMSD trajectories showed that all ligand-bound complexes stabilized within 20 ns, with reduced deviations compared to the apo structure. Notably, ZINC3870414 and ZINC8214398 demonstrated early and sustained convergence, consistent with effective engagement at the ATP-binding site [36,37]. RMSF analyses revealed suppressed flexibility around key catalytic residues, including GLU105, MET107, and ASP178, indicating localized rigidity induced by ligand binding. The ligand-bound systems generally exhibited dampened fluctuations around these residues, indicative of enhanced local rigidity and sustained binding, a feature of kinase inhibition [38]. Rg analysis supported these findings, with the ligand-bound systems maintaining lower and more consistent compactness profiles than the apo protein. These results support the view that ligand binding stabilizes the protein core and limits global conformational drift, as observed in other structure-based kinase studies [36,39]. Principal component analysis further demonstrated a redistribution of dominant motions toward more constrained regions, particularly the hinge and activation loop. The reduced variance in principal components among ligand-bound states suggests a narrowing of conformational space, characteristic of selective inhibitor binding [40]. ZINC31155769 exhibited the most restricted motion profile, aligning with its low RMSD and compact Rg values. These findings align with earlier observations in ALK and related kinases, where inhibitor binding was shown to reduce structural entropy and promote well-defined active or inactive states [31,41]. These dynamic signatures reduced fluctuation, enhanced compactness, and constrained motion were most pronounced in compounds prioritized through machine learning. This reinforces the contribution of model-guided selection in identifying structurally stable binders.

To further characterize the impact of ligand binding on protein flexibility, we explored collective motions using PCA-based conformational landscape analysis. The apo protein projected a broader distribution of conformers in the PC1–PC2 space, signifying greater conformational heterogeneity and intrinsic flexibility. In contrast, ligand-bound systems, particularly those with ZINC3870414 and ZINC31155769, exhibited more compact clustering, indicative of reduced conformational entropy and stabilization of preferred functional states. These results are consistent with previous studies where ligand association limits the conformational sampling of kinases, often locking the protein into catalytically inert forms [36,39]. Notably, the control inhibitor PHA-E429 and ZINC15657732 also showed defined PCA clusters, further validating their compatibility with the ALK binding pocket. The 3D trajectory morphs illustrated a consistent transition from the initial blue (0 ns) to red (100 ns) conformations, reflecting the time-resolved structural shift in each complex. Compounds such as ZINC28540146 and ZINC4654800 induced moderate shifts localized to regulatory regions like the activation loop and hinge, which are commonly associated with kinase modulation and inhibitor sensitivity [42]. Taken together, these data suggest that while all ligands contribute to conformational restriction, ZINC3870414 and ZINC31155769 elicit the most pronounced stabilization effects, which may underlie their higher binding affinity and target specificity. The limited conformational drift observed is in agreement with previous ALK inhibitor profiling where rigidification of key loops was linked to enhanced selectivity [43]. Overall, these results suggest that while all ligands promote stabilization, compounds like ZINC3870414 and ZINC31155769 exert stronger conformational constraints, possibly contributing to their higher binding affinity and specificity.

Hydrogen bonding is critical for ensuring ligand specificity and sustained receptor engagement. Our simulations revealed that GLU105 consistently formed high-occupancy hydrogen bonds across all ligand-bound complexes, including the control (PHA-E429). Located in the hinge region a well-established anchoring site for kinase inhibitors GLU105 maintained persistent interactions that contributed to ligand stabilization throughout the simulation [44,45]. In addition to GLU105, residues such as MET107, ASP178, and GLY177 frequently contributed to sustained interactions. These results align with crystallographic and mutagenesis studies identifying the hinge and activation loop as interaction hotspots in ALK and other kinases [46,47]. Notably, ZINC3870414 and ZINC31155769 each formed multiple long-lived hydrogen bonds within these domains, supporting their high binding affinity and structural stability as observed in RMSD and energy analyses. Importantly, while each compound demonstrated a unique pattern of secondary contacts (ALA34, GLU75, HIE32), the overlap in key anchor residues underscores a conserved binding mechanism. This conserved interaction core may confer resilience to point mutations, consistent with the strategy employed in designing next-generation ALK inhibitors that retain potency against resistant variants [48]. Our hydrogen bonding analysis confirms that high-affinity ligands share a common interaction core, led by GLU105 and MET107, while variations in peripheral contacts fine-tune the binding profile. These interaction patterns provide a structural rationale for the ligand-specific differences observed in binding energetics and conformational stability.

To investigate how ligand binding influences intramolecular communication, residue interaction networks were constructed from dynamic cross-correlation matrices. In the apo form, the network path involved a broad distribution of residues, including Gly10–Gly12, Asp254–Ala260, and intermediate contacts such as Tyr201 and Thr247. This pattern reflects a dispersed and less efficient communication route, which is expected in the absence of ligand stabilization. Upon ligand binding, interaction networks became more focused, with communication paths concentrating around residues proximal to the active site. ZINC3870414 and ZINC8214398 showed similar behavior, converging communication through Gly10, residues 120–128, and Arg199–Val200. These nodes are in close spatial proximity to the active site, suggesting that ligand binding enhanced internal coordination within the protein. ZINC15657732 and ZINC28540146 also redirected the network to residues 234–244, mimicking the path observed in 2XBA. ZINC3870414 and ZINC8214398 exhibited similar network narrowing, channeling communication through Gly10, residues 120–128, and Arg199–Val200 regions adjacent to the ATP-binding pocket. In contrast, ZINC4654800 routed its communication via more distal residues, such as Pro307 and Glu130, potentially reflecting a distinct stabilization mode with broader influence on protein dynamics. Together, these results suggest that ligand-induced reorganization of residue networks may reinforce structural cohesion, contributing to the enhanced stability observed in PCA and RMSD analyses. This shift from distributed to streamlined communication may underline enhanced binding stability and functional inhibition.

Binding free energy estimates from MM-GBSA analysis offered further support for the stability and potential efficacy of the ligand–protein complexes. All tested compounds displayed favorable total binding free energies, with values ranging from −36.57 to −46.18 kcal/mol. ZINC8214398 and ZINC3870414 yielded the most negative ΔG_total_ values, closely followed by ZINC15657732 and ZINC4654800. These values indicate strong thermodynamic favorability for complex formation, largely driven by van der Waals and electrostatic contributions. Notably, the reference inhibitor (2XBA) exhibited a less favorable ΔG_total_ (−36.57 kcal/mol), suggesting that several screened compounds may offer superior stabilization. Among them, ZINC3870414 displayed a ΔG_total_ of −46.02 kcal/mol and formed persistent hydrogen bonds with key residues such as Glu105, Asp178, and Glu75. This is consistent with its dynamic performance across multiple analyses, including compact conformational distribution, focused residue communication, and stable hydrogen bonding. These energetic and structural observations collectively point to ZINC3870414 and ZINC8214398 as promising chemical starting points for inhibitor development. Their capacity to stabilize the kinase domain through energetically favorable and structurally coherent interactions highlights their therapeutic potential. The convergence of performance across docking, dynamics, and energy-based metrics supports the utility of machine learning-assisted prioritization for identifying high-value chemical scaffolds.

Taken together, the integrated results from virtual screening, molecular dynamics simulations, interaction mapping, and binding free energy analyses converge to support the potential of selected hits, particularly ZINC3870414 and ZINC8214398, as credible ALK inhibitors. These compounds consistently demonstrated stable binding, maintained interactions with conserved catalytic residues, and induced conformational effects comparable to or exceeding those of the reference inhibitor. The inclusion of machine learning in the screening pipeline enhanced early-stage compound prioritization, allowing identification of ligands with favorable dynamic, structural, and energetic characteristics. While further experimental validation is required, this computational framework provides a robust starting point for advancing these compounds into biochemical and cellular evaluation. The findings highlight the added value of dynamic and network-based assessments in complementing conventional structure-based screening approaches.

### 3.1. Study Strengths

Our study presents an integrated computational framework that combines supervised machine learning, structure-based virtual screening, molecular dynamics simulations, and network-level residue analysis. A key strength lies in the use of multiple, orthogonal metric binding energetics, conformational dynamics, and communication efficiency to consistently prioritize high-affinity compounds. The inclusion of data-driven model selection enhanced the specificity of compound ranking, while dynamic and structural validation ensured robustness of predictions. The convergence of results across these distinct layers supports the reliability of the identified candidates as potential ALK inhibitors.

### 3.2. Future Directions

Future efforts of our study will focus on expanding the compound screening to include ALK variants associated with resistance, particularly those involving point mutations in the kinase domain. Modeling of mutant-binding scenarios will help assess scaffold flexibility and potential for resistance evasion. Additionally, top-ranked compounds will be subjected to experimental validation through biochemical assays and cellular models. These steps will be critical to establish the translational relevance of the computational predictions and advance lead compounds toward preclinical development.

## 4. Methodology

### 4.1. Study Objective

This study aimed to identify new small-molecule inhibitors of the ALK domain by using the structural diversity of natural product-like compounds. We applied an integrated computational strategy combining structure-based virtual screening, supervised machine learning, molecular dynamics simulations, and residue network analysis. The goal was to prioritize compounds with favorable binding profiles and physicochemical properties for further investigation as potential scaffolds in the early-stage discovery of ALK-targeted therapies.

**Figure 9:**
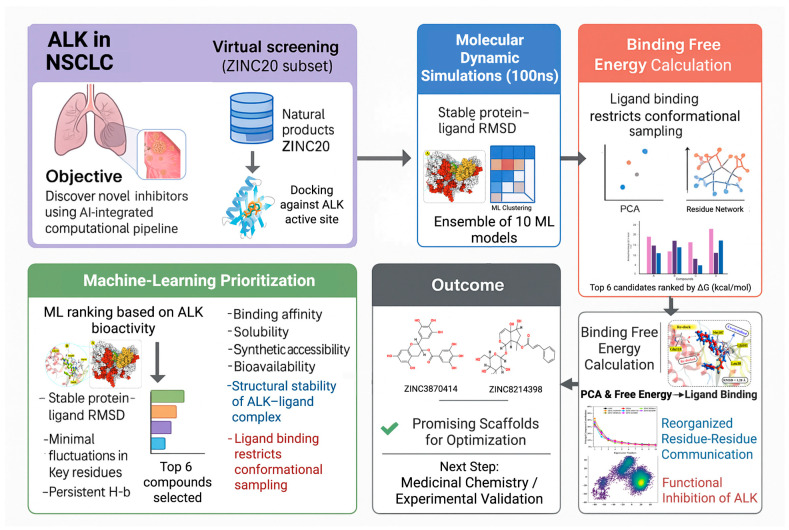
An overview of the AI-assisted drug discovery pipeline used to identify novel ALK inhibitors in NSCLC. The workflow integrates virtual screening of the ZINC20 natural product subset, docking against the ALK domain, ensemble machine learning prioritization, molecular dynamics simulations, and binding free energy calculations. Candidate compounds were evaluated for structural stability, binding energetics, and pharmacokinetic properties. Top-scoring hits, including ZINC3870414 and ZINC8214398, were identified as promising scaffolds for further optimization and experimental validation.

Figure 9 provides an overview of the computational workflow.

### 4.2. Protein Crystal Coordinates Selection

The crystal structure of human ALK in complex with the PHA-E429 inhibitor (PDB ID: 2XBA; resolution: 1.95 Å) was retrieved from the Protein Data Bank (https://www.rcsb.org/structure/2XBA, accessed on 4 June 2025) [26]. This structure was selected based on its high resolution and the presence of a co-crystallized ligand, indicating a well-defined binding site. Protein preparation was performed using UCSF ChimeraX (version 1.10) (https://www.cgl.ucsf.edu/chimerax/) [49]. All crystallographic water molecules were removed. Missing residues were modeled using the ModRefiner server (http://zhanggroup.org/ModRefiner/, accessed on 4 June 2025) [50], which rebuilds incomplete protein regions while maintaining stereochemical integrity. Hydrogen atoms were added, and the protonation states of ionizable residues were adjusted to pH 7.4 using PDB2PQR (version 3.7.1) (https://server.poissonboltzmann.org/pdb2pqr, accessed on 4 June 2025) [51] and PROPKA (https://www.ddl.unimi.it/vegaol/propka.htm, accessed on 4 June 2025) for pKa estimation [52]. Missing force field parameters were assigned using the AmberTools24 suite [53], and applied the ff19SB force field [54]. The charges were assigned to optimize hydrogen bonding networks, and correcting side-chain orientations where necessary. The final prepared protein structure was saved in PDBQT format using AutoDockTools (version 1.5.7) for downstream docking simulations.

### 4.3. Compounds Selection and Preparation

A subset of natural product-like compounds was obtained from the ZINC20 database (https://zinc20.docking.org, accessed on 10 June 2025), consisting of over 220,000 commercially available molecules. Compounds were downloaded in SMILES format and processed to compute molecular descriptors consistent with those used for model development. Binary molecular fingerprints were calculated using PaDEL-Descriptor, applying the same configuration files and preprocessing settings as used in model training. Ten fingerprint types were generated: AtomPairs2D, AtomPairs2DCount, EState, CDK, CDKextended, CDKgraphonly, KlekotaRoth, KlekotaRothCount, MACCS, and PubChem. Descriptor calculation was standardized across all compounds to ensure compatibility with downstream predictions.

### 4.4. Data Retrieval

The dataset used in this study was obtained from the ChEMBL database (https://www.ebi.ac.uk/chembl/, accessed on 13 June 2025) (ChEMBL ID: CHEMBL279). Bioactivity data corresponding to *IC*_50_ values for ALK inhibitors were retrieved using the ChEMBL web services client in Python (version 3.10). The query filtered for compounds with reported *IC*_50_ values in nanomolar units and a defined target relationship. The chemical structures were retrieved as SMILES strings. Initial filtering removed entries lacking valid bioactivity measurements. The *IC*_50_ values were converted to pIC_50_ values using the transformation (Equation (1)). Compounds were classified as active (pIC_50_ > 6), inactive (pIC_50_ < 5), or intermediate (5 ≤ pIC_50_ ≤ 6). Only compounds classified as active or inactive were used for subsequent analysis.

(1) $${log}_{10}\left({IC}_{50}\;inmolarunits\right)$$

### 4.5. Molecular Descriptor Calculation

Molecular descriptors were computed using PaDEL-Descriptor (v2.21) through the padelpy Python wrapper [55]. The input dataset comprised 1259 compounds, each represented by its SMILES string and ChEMBL identifier. Twelve fingerprint types were calculated: AtomPairs2D, AtomPairs2DCount, EState, CDK, CDKextended, CDKgraphonly, KlekotaRoth, KlekotaRothCount, MACCS, PubChem, Substructure, and SubstructureCount. Descriptors were calculated using XML configuration files from the PaDEL repository. Standard preprocessing included aromaticity detection, nitro group normalization, tautomer standardization, and salt removal. Following generation, each descriptor set was processed to retain only binary features (0/1). Features with near-zero variance (threshold < 1 × 10^−5^) were removed using a VarianceThreshold filter. Redundant binary features were filtered using pairwise Tanimoto similarity (threshold > 0.9) to reduce dimensionality while preserving chemical diversity. The final descriptor set for each fingerprint type was merged with corresponding pIC_50_ values for downstream modeling.

### 4.6. Model Training and Evaluation

Eleven machine learning classifiers were trained using scikit-learn and LightGBM: Random Forest, XGBoost, LightGBM, Support Vector Classifier (SVC), Gradient Boosting, AdaBoost, Extra Trees, Ridge Classifier, Logistic Regression, and ElasticNet. Additionally, a custom artificial neural network (ANN) was implemented separately using PyTorch (version 2.0.1). [56]. For each descriptor set, compounds were labeled as active (pIC_50_ ≥ 6) or inactive (pIC_50_ < 6). The dataset was randomly split into training and validation sets (80:20 ratio), and each model was trained and evaluated over 100 iterations. Performance was assessed using accuracy, AUC, F1-score, recall, specificity, and precision. The ANN model consisted of three fully connected layers (input–128–64–1) with ReLU activations and sigmoid output, trained using binary cross-entropy loss and the Adam optimizer for 100 epochs. Metrics were averaged across all runs to capture the stability and generalization of each model. ROC curves and confusion matrices were generated from the first iteration. The ANN model was implemented and saved using PyTorch, while the other models were developed using scikit-learn. AUC and accuracy averages were used to rank model-descriptor combinations, with results visualized through heatmaps and clustering.

### 4.7. Virtual Screening of Natural Products

The top-performing model was applied to screen natural product compounds from the ZINC20 natural product database. Descriptors for the screening set were calculated using the same fingerprint method as used during model training. The model was selected based on highest average AUC and accuracy. The artificial neural network model was implemented and saved using PyTorch, while all other machine learning models used in the final analysis were developed and managed using scikit-learn with joblib. Predictions were generated as probability scores of compound activity. Compounds with predicted probabilities ≥ 0.7 were retained. The top 50 scoring compounds were also identified. For all predictions, compound metadata and descriptor values were retained for downstream analysis.

### 4.8. Molecular Docking Simulation

Molecular docking was performed on the top 50 compounds identified through AI-based virtual screening of the ZINC20 natural product subset. Compounds were prepared by converting SMILES to SDF format using RDKit [57], generating 3D conformations with the ETKDG method, and refining structures using Open Babel. Protonation states were adjusted to pH 7.4, and hydrogen atoms were added. Final structures were converted to PDBQT format using AutoDockTools. Docking was carried out against the PHA-E429 binding site of the human ALK protein using AutoDock Vina (version 1.1.2) [58]. The docking grid was restricted to the active site region. Ligands were ranked based on predicted binding affinities, and root-mean-square deviation (RMSD) values were used to assess pose consistency. Top-scoring compounds were retained for further evaluation.

### 4.9. Prediction of Pharmacokinetic and Physicochemical Properties

Pharmacokinetic and physicochemical properties were evaluated for the top-ranked compounds retained after molecular docking. Key descriptors were predicted using SwissADME (http://www.swissadme.ch/, accessed on 16 June 2025), including molecular weight, lipophilicity, aqueous solubility, hydrogen bonding potential, and oral bioavailability. These parameters were estimated using both experimental data and predictive models integrated within the SwissADME platform. Compounds with unfavorable pharmacokinetic profiles or non-compliant physicochemical properties were excluded from further consideration to prioritize candidates with acceptable drug-like characteristics and systemic exposure potential.

### 4.10. Molecular Dynamic Simulations

Molecular dynamics (MD) simulations were carried out using the AMBER 24 (https://ambermd.org/) [53] software suite to evaluate the structural stability of protein–ligand complexes. Protein atoms were assigned parameters using the ff19SB force field [54], while ligand topologies were generated with GAFF2 [59]. Partial atomic charges for ligands were derived using the AM1-BCC method following geometry optimization via Gaussian [59]. Complexes were prepared using the tLEaP module and solvated in an octahedral OPC water box with a 12 Å buffer. Systems were neutralized and ionized to a physiological salt concentration (0.15 M NaCl) with Na^+^ and Cl^−^ counterions [60]. Energy minimization was conducted in two phases: a restrained minimization (10 kcal·mol^−1^·Å^−2^) using 50,000 steps of steepest descent, followed by an unrestrained 20,000-step conjugate gradient run. The systems were heated gradually from 0 to 300 K under constant volume (NVT) for 500 ps using a Langevin thermostat (1 ps^−1^ collision frequency) [61]. This was followed by 500 ps of pressure equilibration under constant pressure (NPT) using a Monte Carlo barostat (2 ps relaxation time), and an additional 1 ns of equilibration without restraints. Production simulations were run for 100 ns using the pmemd.CUDA module to use GPU acceleration. Long-range electrostatics were treated with the Particle Mesh Ewald (PME) method, and an 8 Å cutoff was applied to non-bonded interactions. Bond constraints involving hydrogen atoms were enforced using SHAKE, and coordinates were saved every 10 ps for analysis.

### 4.11. Protein Stability and Flexibility Analysis

The structural stability and flexibility of the ALK–ligand complexes were examined using the CPPTRAJ module in AMBER 24 [62]. RMSD was calculated for the Cα atoms of ALK and the heavy atoms of the ligands across the 100 ns simulation, using the minimized structure as reference. Root mean square fluctuation (RMSF) was computed to assess residue-level flexibility and identify regions involved in ligand binding. The radius of gyration (Rg) was analyzed to evaluate overall protein compactness and structural integrity during the simulation. These metrics allowed for a comparative assessment of conformational dynamics across all complexes [62].

### 4.12. Principal Component Analysis (PCA)

Principal component analysis (PCA) was used to investigate large-scale collective motions in the ALK–ligand complexes during molecular dynamics simulations. A covariance matrix of Cα atomic displacements was constructed, and eigenvector decomposition was applied to extract dominant modes of motion. The first two principal components (PC1 and PC2), which captured the majority of structural variance, were analyzed to characterize conformational shifts. Trajectories were projected onto these components to visualize the conformational landscape sampled by each complex, to capture the ligand-induced dynamic transitions and protein flexibility [62].

### 4.13. Hydrogen Bond Analysis

Hydrogen bonding patterns between ALK and the bound ligands were analyzed across the 100 ns simulation using the CPPTRAJ module. A hydrogen bond was defined by a donor–acceptor distance ≤ 3.5 Å and a donor–hydrogen–acceptor angle ≥ 120°. The occupancy of each hydrogen bond was calculated to evaluate its persistence over time. Particular attention was given to interactions within the active site, especially those involving catalytically relevant residues. High-occupancy hydrogen bonds were identified as key contributors to the stability of the protein–ligand complexes [62].

### 4.14. Dynamic Cross-Correlation Network Analysis

To evaluate residue-residue communication within the protein structure, the dynamic cross-correlation matrix (DCCM) was calculated based on the Cα atom fluctuations over the course of the simulation. The resulting matrix was used to construct an undirected residue interaction network. Residues were represented as nodes, and edges were established between pairs exhibiting a cross-correlation coefficient of 0.5 or higher. Edge weights were defined as one minus the absolute correlation value, allowing the identification of the shortest communication paths between selected residue pairs [62]. The resulting network was further analyzed to trace potential identification of residue networks that may support long-range signal transmission or functional coupling between catalytic activity and protein interaction.

### 4.15. Binding Free Energy Calculations (MM/GBSA)

Binding free energies of the ALK–ligand complexes were calculated using the molecular mechanics/generalized Born surface area (MM/GBSA) method implemented in AMBER 24. Calculations were performed on snapshots extracted from the final 25 ns of each trajectory to ensure adequate statistical sampling. The binding free energy (Δ*G_bind_*) was estimated using Equation (2) [62]:

(2) $$\Delta G_{bind}=\;{\Delta G}_{R+L}-\left(\Delta G_{R}+{\Delta G}_{L}\right)$$

where G_complex_, G_protein_, and G_ligand_ represent the free energies of the complex, isolated protein, and isolated ligand, respectively.

The total free energy was decomposed into key components, bonded interactions (*E_bond_*), van der Waals interactions (*E_vdW_*), electrostatic energy (*E_elec_*), polar solvation energy (*G_GB_*, using the GB model), and non-polar solvation energy (*G_SASA_*, using the SASA model). Entropic contributions (*T*Δ*S*) were not included due to computational constraints and their limited influence on relative binding estimates (Equation (3)) [62].

(3) $$G=E_{bond}+E_{VDW}+E_{elec}+G_{GB}+G_{SA}-TS_{S}$$

Final Δ*G_bind_* values were reported in kcal/mol and used to compare binding affinities across all complexes.

### 4.16. Data Analysis

Trajectory processing was carried out using the CPPTRAJ module in AMBER 24. Structural visualization and interaction mapping were performed with VMD (version 2.0) (https://www.ks.uiuc.edu/Research/vmd/) [63] and PyMOL (version 3.1.3) (https://www.pymol.org/) [64]. Quantitative analysis included RMSD, RMSF, radius of gyration, principal component projections, and hydrogen bond occupancies were visualized using Matplotlib (version 3.7.1) [65].

## 5. Conclusions

Aberrant ALK signaling is implicated in the pathogenesis of multiple cancers, including non-small cell lung cancer, making it a well-established target for therapeutic intervention. Here, we employed a multi-step computational approach combining structure-based virtual screening, molecular docking, molecular dynamics simulations, and free energy calculations to identify potential ALK inhibitors from a library of lead-like natural products. Machine learning models trained on curated inhibitor datasets were integrated into the screening workflow to refine compound prioritization and enhance early-stage selection accuracy. Among the top-ranked candidates, ZINC3870414 and ZINC8214398 consistently exhibited stable binding, favorable energetic profiles, and persistent engagement of key catalytic residues. Analyses of conformational dynamics and communication networks further supported their capacity to stabilize functionally relevant ALK states. These findings highlight ZINC3870414 and ZINC8214398 as computationally prioritized scaffolds for future investigation and support the utility of integrating machine learning with dynamic simulation and network-based descriptors in early-stage kinase inhibitor discovery.

## 6. Limitations

While the results provide a detailed structural and dynamical assessment of ligand interactions with ALK, they reflect a simplified model system. Biological factors such as metabolism, cell permeability, and systemic distribution were not addressed. Future experimental work, including in vitro and in vivo assays, will be needed to confirm inhibitory activity and therapeutic relevance. Still, the present analysis offers a strong rationale for prioritizing these candidates for further validation.

## Figures and Tables

**Table 1:** Docking results of selected ZINC20 natural product-like compounds compared to the reference inhibitor PHA-E429. The table lists docking scores (kcal/mol) and RMSD values (Å) for the top six candidates and the control compound co-crystallized in the ALK structure (PDB ID: 2XBA). n the chemical structures, red indicates oxygen atoms and hydroxyl groups, and blue indicates nitrogen atoms.

ZINC-ID	2D Structure	Docking Score	RMSD (Å)
ZINC3870414	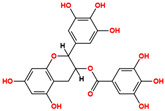	−10.31	1.8
ZINC31155769	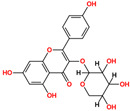	−9.46	1.5
ZINC15657732	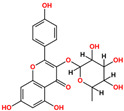	−9.14	2.7
ZINC8214398	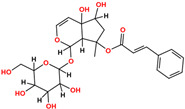	−9.05	2.0
ZINC28540146	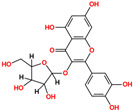	−8.90	2.6
ZINC4654800	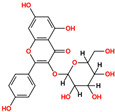	−8.90	1.2
2XBA (PHA-E429)	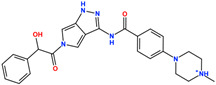	−8.78	1.2

**Table 2:** MM-GBSA binding free energy components (kcal/mol) for ALK-ligand complexes, including van der Waals (ΔE_VDW_), electrostatic (ΔE_EL_), solvation (ΔE_GB_, ΔE_SASA_), gas phase (ΔG_GAS_), and total binding free energy (ΔG_TOTAL_).

Complex		MM-GBSA Calculations (All Unit’s kcal/mol)断Differences (Complex–Receptor–Ligand)					
	ΔE_VDW_	ΔE_EL_	ΔE_GB_	ΔE_SASA_	ΔG_GAS_	ΔG_SOLV_	ΔG_TOTAL_
2XBA	−49.50 ± 0.44	−117.11 ± 0.11	136.11 ± 0.43	−6.06 ± 0.011	−166.61 ± 0.47	130.04 ± 0.43	−36.57 ± 0.12
ZINC3870414	−45.36 ± 0.12	−78.26 ± 0.20	85.53 ± 0.10	−7.93 ± 0.005	−123.62 ± 0.17	77.59 ± 0.10	−46.02 ± 0.12
ZINC31155769	−38.95 ± 0.16	−49.32 ± 0.37	54.63 ± 0.20	−6.05 ± 0.21	−90.28 ± 0.44	48.58 ± 0.20	−39.69 ± 0.36
ZINC15657732	−47.50 ± 0.10	40.04 ± 0.43	52.72 ± 0.31	−6.06 ± 0.011	−87.55 ± 0.42	46.65 ± 0.30	−40.89 ± 0.16
ZINC8214398	−42.26 ± 0.12	−28.93 ± 0.30	40.05 ± 0.24	−5.03 ± 0.012	−81.20 ± 0.31	35.01 ± 0.24	−46.18 ± 0.12
ZINC28540146	−37.21 ± 0.11	−51.00 ± 0.31	54.46 ± 0.18	−6.10 ± 0.010	−88.22 ± 0.29	48.35 ± 0.17	−39.86 ± 0.17
ZINC4654800	−40.03 ± 0.13	−58.34 ± 0.74	63.65 ± 0.62	−5.84 ± 0.021	−98.37 ± 0.78	57.81 ± 0.61	−40.56 ± 0.23

ΔE_VDW_, van der Waals free energy: ΔE_EL_, electrostatic free energy: ΔE**_G_**_B_, the polar component of solvation-free energy: ΔE_SASA_, non-polar components of solvation energy: ΔG_GAS_, binding free energy without solvent: ΔG_SOLV_, binding free energy with solvent: ΔG_TOTAL_, total binding free energy**.**

## Data Availability

Data is contained within the article or Supplementary Material.

## Supplementary Materials

The following supporting information can be downloaded at: https://www.mdpi.com/article/10.3390/ph18081178/s1, Table S1: Performance of 10 machine learning algorithms across 10 molecular fingerprints ranked by average AUC and Accuracy; Table S2: Molecular interactions of top compounds and PHA-E429 in the ALK active site; Table S3: Physicochemical properties of six shortlisted compounds (SwissADME); Table S4: ADME profiles of six shortlisted compounds (SwissADME); Table S5: Hydrogen bond profiles from MD simulations for selected ligands and reference compound (2XBA); Figure S1: Boxplots of LogP, MW, NumHDonors, NumHAcceptors by activity class; Figure S2: Performance metrics for RandomForest, XGBoost, and PyTorchANN across fingerprints; Figure S3: ROC curves for AtomPairs2D fingerprint models; Figure S4: ROC curves for AtomPairs2D_FingerPrinter descriptor; Figure S5: ROC curves for Estate_FingerPrinter models; Figure S6: ROC curves for FingerPrinter descriptor; Figure S7: ROC curves for KlekotaRoth_FingerPrinter models; Figure S8: ROC curves for GraphOnly_FingerPrinter models; Figure S9: ROC curves for MACCS_FingerPrinter models; Figure S10: ROC curves for PubChem_FingerPrinter-based models; Figure S11: ROC curves for Substructure_FingerPrinter models; Figure S12: Confusion matrices for AtomPairs2D_fingerPrintCount; Figure S13: Confusion matrices for AtomPairs2D_FingerPrinter descriptors; Figure S14: Confusion matrices for Estate_FingerPrinter descriptor; Figure S15: Confusion matrices for Extended_FingerPrinter descriptor; Figure S16: Confusion matrices for FingerPrinter descriptor; Figure S17: Confusion matrices for GraphOnly_FingerPrinter representations; Figure S18: Confusion matrices for KlekotaRoth_FingerPrinter; Figure S19: Confusion matrices for MACCS_FingerPrinter; Figure S20: Confusion matrices for Pubchem_FingerPrinter; Figure S21: Confusion matrices for Substructure_FingerPrinter descriptor.

## Abbreviations

The following abbreviations are used in this manuscript:

ALK	Anaplastic lymphoma kinase
NSCLC	Non-small cell lung cancer
TKI	Tyrosine kinase inhibitor
PCA	Principal component analysis
MD	Molecular dynamics
RMSD	Root-mean-square deviation
RMSF	Root-mean-square fluctuation
MM/GBSA	Molecular mechanics/generalized Born surface area
AUC	Area under the curve
ROC	Receiver operating characteristic
ADMET	Absorption, distribution, metabolism, excretion, and toxicity
