# Supplementary material for: Machine Learning and Integrative Structural Dynamics Identify Potent ALK Inhibitors from Natural Compound Libraries
Source: Pharmaceuticals (Basel). 2025 Aug 10;18(8):1178. doi: 10.3390/ph18081178 (PMC12389032; doi:10.3390/ph18081178)
Supplement: Supplementary file 1 [file pharmaceuticals-18-01178-s001.zip › pharmaceuticals-3744152-supplementary.pdf]

Supporting information

Machine Learning and Integrative Structural Dynamics Identify Potent ALK Inhibitors from Natural Compound Libraries

Rana Alateeq[1*]

[1]Department of Medical Laboratories, College of Applied Medical Sciences, Qassim University, Burydah 51452, Saudi Arabia.

Correspondence: Rana Alateeq, Department of Medical Laboratories, College of Applied Medical Sciences, Qassim University, Burydah 51452, Saudi Arabia, R.ALATEEQ@qu.edu.sa

Table of contents

Table S1. Comprehensive evaluation of 10 machine learning algorithms across 10 molecular fingerprint descriptors for bioactivity prediction. Performance metrics include Accuracy, Area Under the Receiver Operating Characteristic Curve (AUC), F1 score, Recall, Specificity, and Precision. Models are ranked by the average of AUC and Accuracy (AUC_Acc_Avg), used as a balanced metric for overall performance. 25

Table S2. Molecular interactions of top-ranked compounds and the reference ligand (PHA-E429) within the ALK active site. Hydrogen bond donors (HBD), hydrogen bond acceptors (HBA), ionic contacts, and π-H interactions are listed along with their respective interaction distances and estimated binding energies. 29

Table S3. Physicochemical properties of shortlisted six compounds calculated using the SwissADME server. 30

Table S4. Absorption and distribution excretion profile of shortlisted six compounds computed via SwissADME server. 30

Table S5. Hydrogen bond interaction profile of selected ligands and the reference compound (2XBA) within the ALK active site, based on molecular dynamics simulations. For each interaction, the hydrogen bond acceptor and donor atoms, average bond life (occupancy %), distance (Å), and angle (˚) are listed. 31

Figure S1. Physicochemical properties by activity class. Boxplots of LogP, molecular weight (MW), number of hydrogen bond donors (NumHDonors), and acceptors (NumHAcceptors) for active, inactive, and intermediate compounds. Active compounds generally show lower MW and fewer hydrogen bond donors than inactive. 4

Figure S2. Performance metrics (Accuracy, AUC, F1, Recall, Specificity, and Precision) for RandomForest, XGBoost, and PyTorchANN models across ten molecular fingerprint descriptors. Each subplot summarizes the classification efficacy of a given descriptor-model combination,

providing insight into model robustness and descriptor informativeness in the context of binary activity prediction. 5
Figure S3. Receiver operating characteristic (ROC) curves for AtomPairs2D fingerprint models using different classifiers. All models showed consistent classification performance, with AUC values ranging from 0.86 to 0.92. 6
Figure S4. ROC curves for AtomPairs2D_FingerPrinter descriptor across eleven classifiers. Top-performing models included XGBoost (AUC = 0.93) and PyTorchANN (AUC = 0.94), indicating high discriminative ability........ 7
Figure S5. ROC curves for Estate_FingerPrinter models. GradientBoosting and XGBoost achieved AUC values of 0.85 and 0.91, respectively. AdaBoost performed comparatively lower (AUC = 0.79). 8
Figure S6. ROC curves for the generic FingerPrinter descriptor across all classifiers. LightGBM, XGBoost, and GradientBoosting yielded the highest AUCs (~0.95), confirming robust predictive performance. 9
Figure S7. ROC curves for models using the KlekotaRoth_FingerPrinter descriptor. XGBoost and LightGBM provided the highest classification accuracy, each with AUCs of 0.95. 10
Figure S8. ROC curves for GraphOnly_FingerPrinter models. LightGBM and XGBoost performed consistently well (AUC = 0.94 and 0.92), with SVC also achieving high accuracy (AUC = 0.92). 11
Figure S9. ROC curves for MACCS_FingerPrinter models. Performance was highest for XGBoost (AUC = 0.96), followed closely by LightGBM and GradientBoosting (AUC = 0.95 and 0.90, respectively). 12
Figure S10. ROC curves for PubChem_FingerPrinter-based models. Most classifiers achieved AUC > 0.90, with the highest observed for XGBoost (AUC = 0.96). 13
Figure S11. ROC curves for Substructure_FingerPrinter models. XGBoost and LightGBM showed AUC values of 0.93 and 0.90, respectively, outperforming other classifiers. 14
Figure S12. Confusion matrices for AtomPairs2D_fingerPrintCount across different machine learning algorithms. 15
Figure S13. Confusion matrices for AtomPairs2D_FingerPrinter descriptors. Models were evaluated on their ability to accurately classify compounds using 2D atom pair fingerprints. 16
Figure S14. Confusion matrices for the Estate FingerPrinter descriptor across all tested models. Estate fingerprints provided varying resolution of bioactivity patterns as reflected in classification outcomes. 17
Figure S15. Confusion matrices for the Extended FingerPrinter descriptor with 10 machine learning classifiers. Performance varied across models, indicating model-dependent effectiveness of the extended fingerprint features. 18
Figure S16. Confusion matrices for the standard FingerPrinter descriptor. These results demonstrate the predictive consistency of this classical representation across most models. 19
Figure S17. Confusion matrices for GraphOnly_FingerPrinter representations. Classification patterns reflect the models' handling of topological graph-based features in bioactivity prediction. 20

Figure S18. Confusion matrices for KlekotaRoth_FingerPrinter, showing strong predictive separability in some models and modest confusion in others, underlining the impact of feature dimensionality. 21
Figure S19. Confusion matrices for MACCS_FingerPrinter across all classifiers. MACCS keys provide compact yet informative features for bioactivity classification. 22
Figure S20. Confusion matrices for Pubchem_FingerPrinter across 10 models. The results reflect how standardized structural keys perform across diverse machine learning settings. 23
Figure S21. Confusion matrices for classification using the Substructure FingerPrinter descriptor across 10 machine learning models. 24

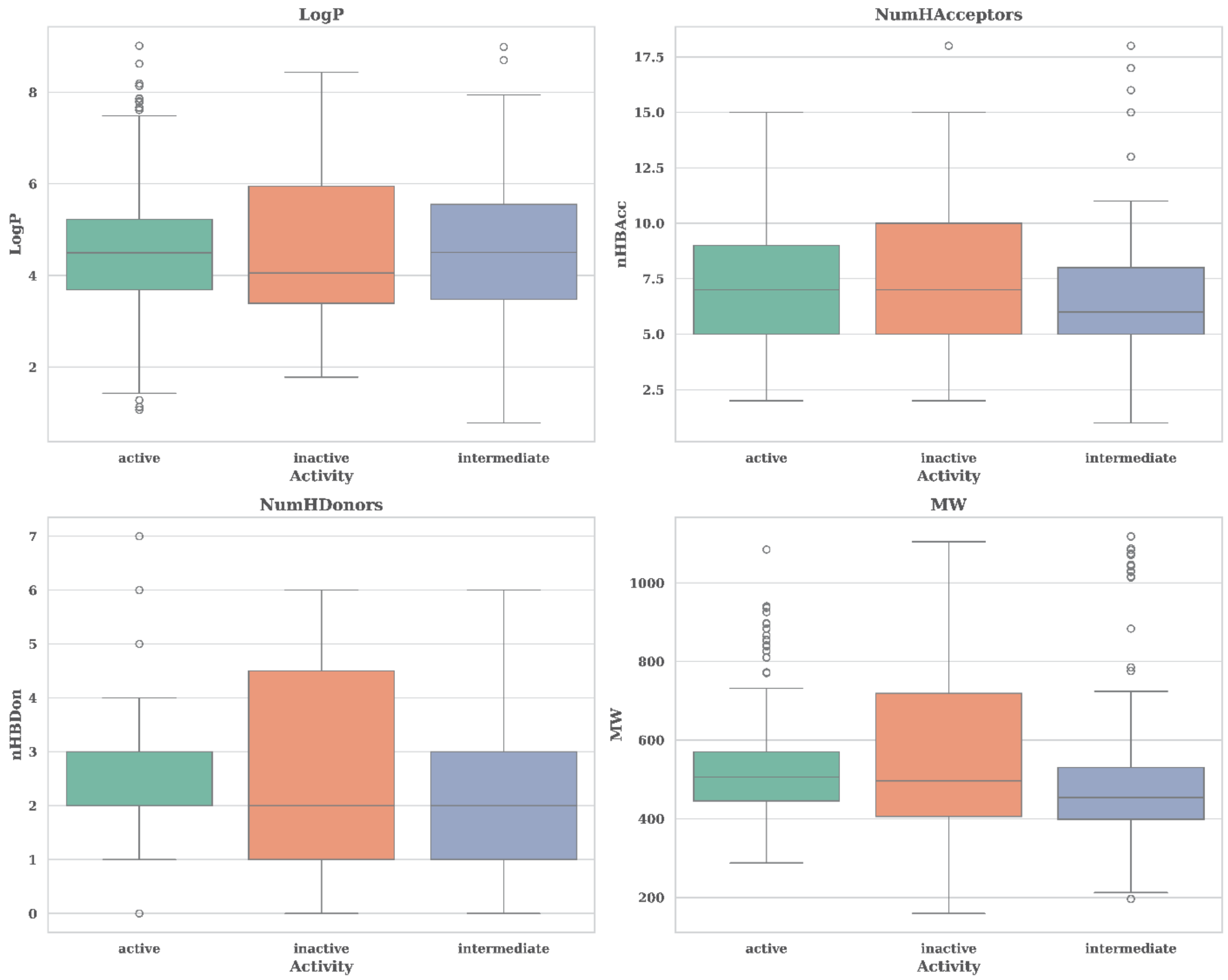

Figure S1. Physicochemical properties by activity class. Boxplots of LogP, molecular weight (MW), number of hydrogen bond donors (NumHDonors), and acceptors (NumHAcceptors) for active, inactive, and intermediate compounds. Active compounds generally show lower MW and fewer hydrogen bond donors than inactive.

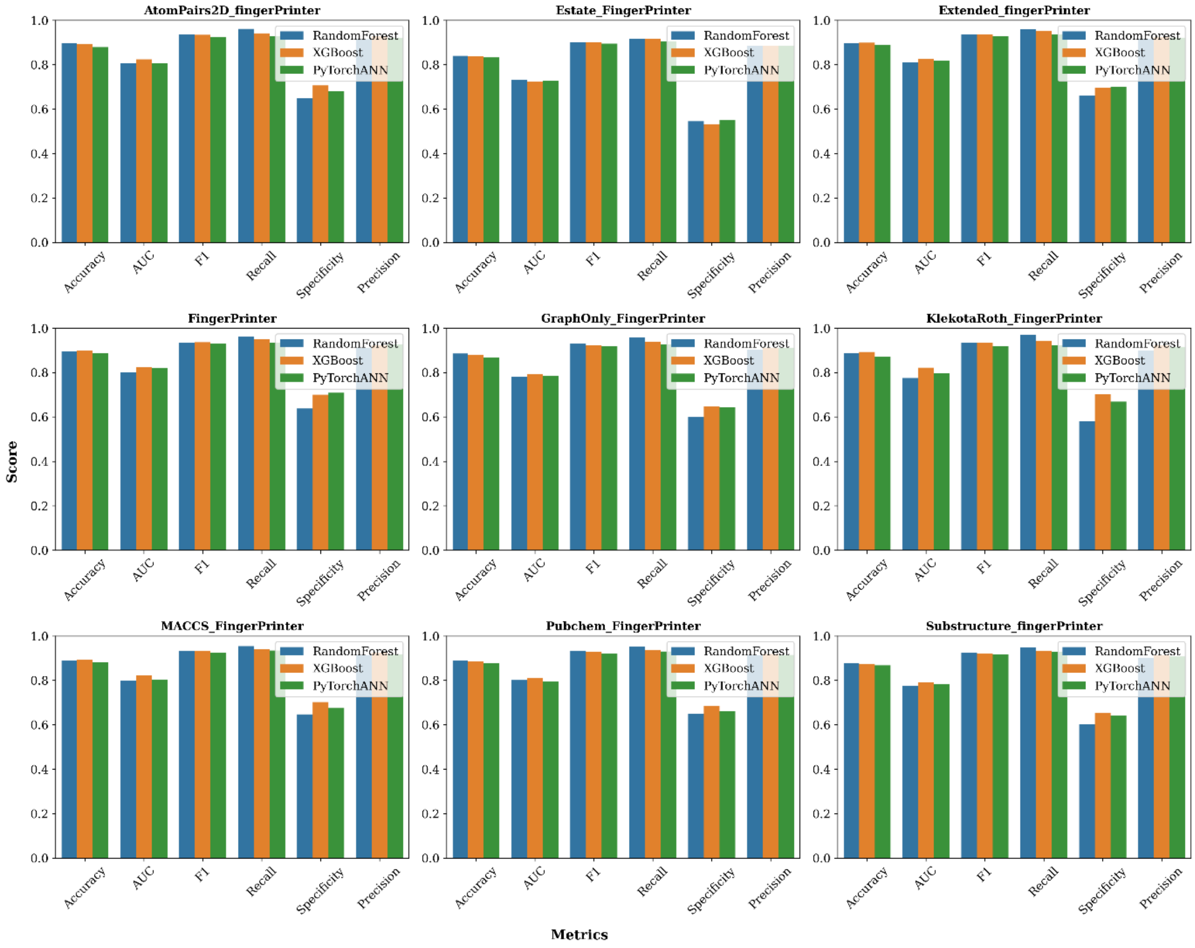

Figure S2. Performance metrics (Accuracy, AUC, F1, Recall, Specificity, and Precision) for RandomForest, XGBoost, and PyTorchANN models across ten molecular fingerprint descriptors. Each subplot summarizes the classification efficacy of a given descriptor-model combination, providing insight into model robustness and descriptor informativeness in the context of binary activity prediction.

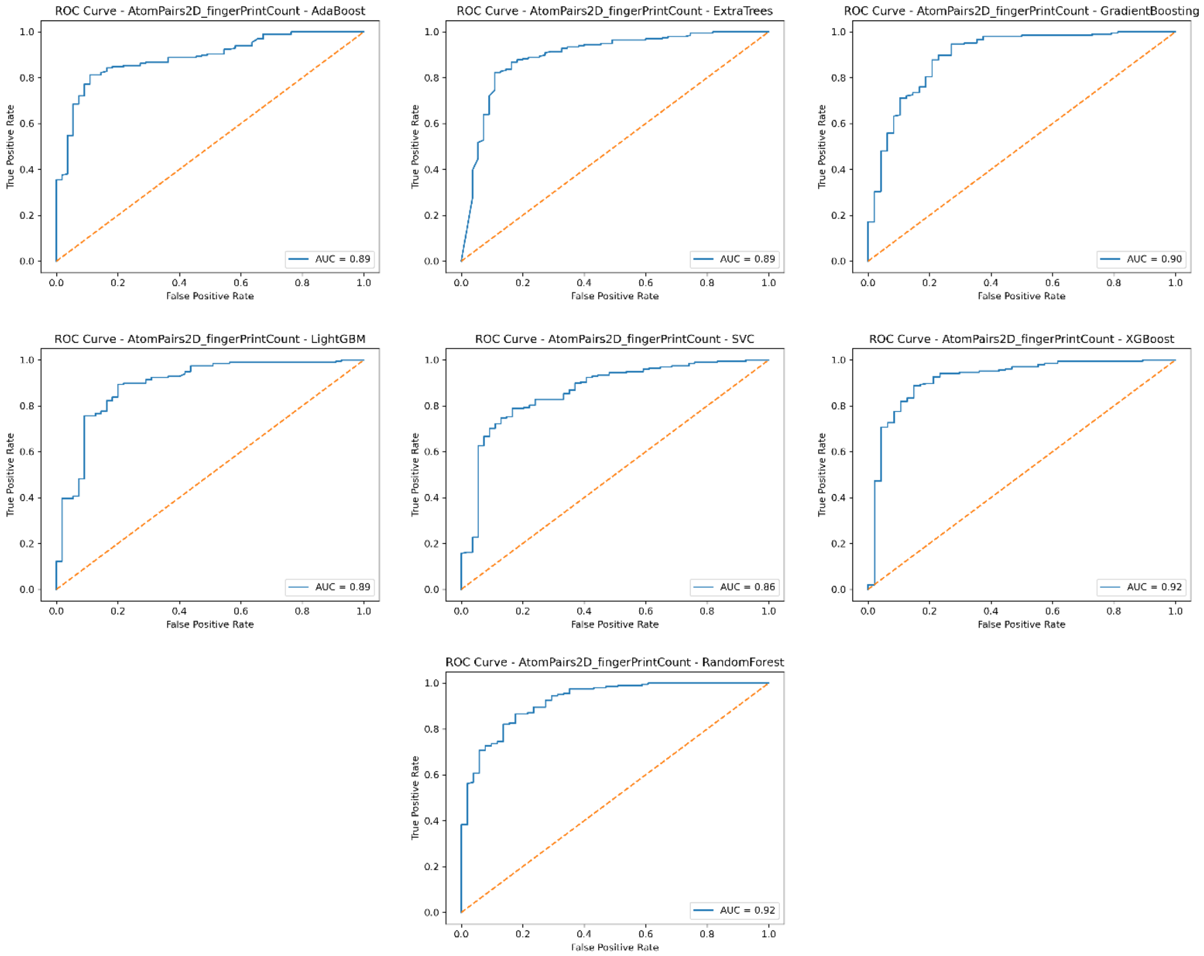

Figure S3. Receiver operating characteristic (ROC) curves for AtomPairs2D fingerprint models using different classifiers. All models showed consistent classification performance, with AUC values ranging from 0.86 to 0.92.

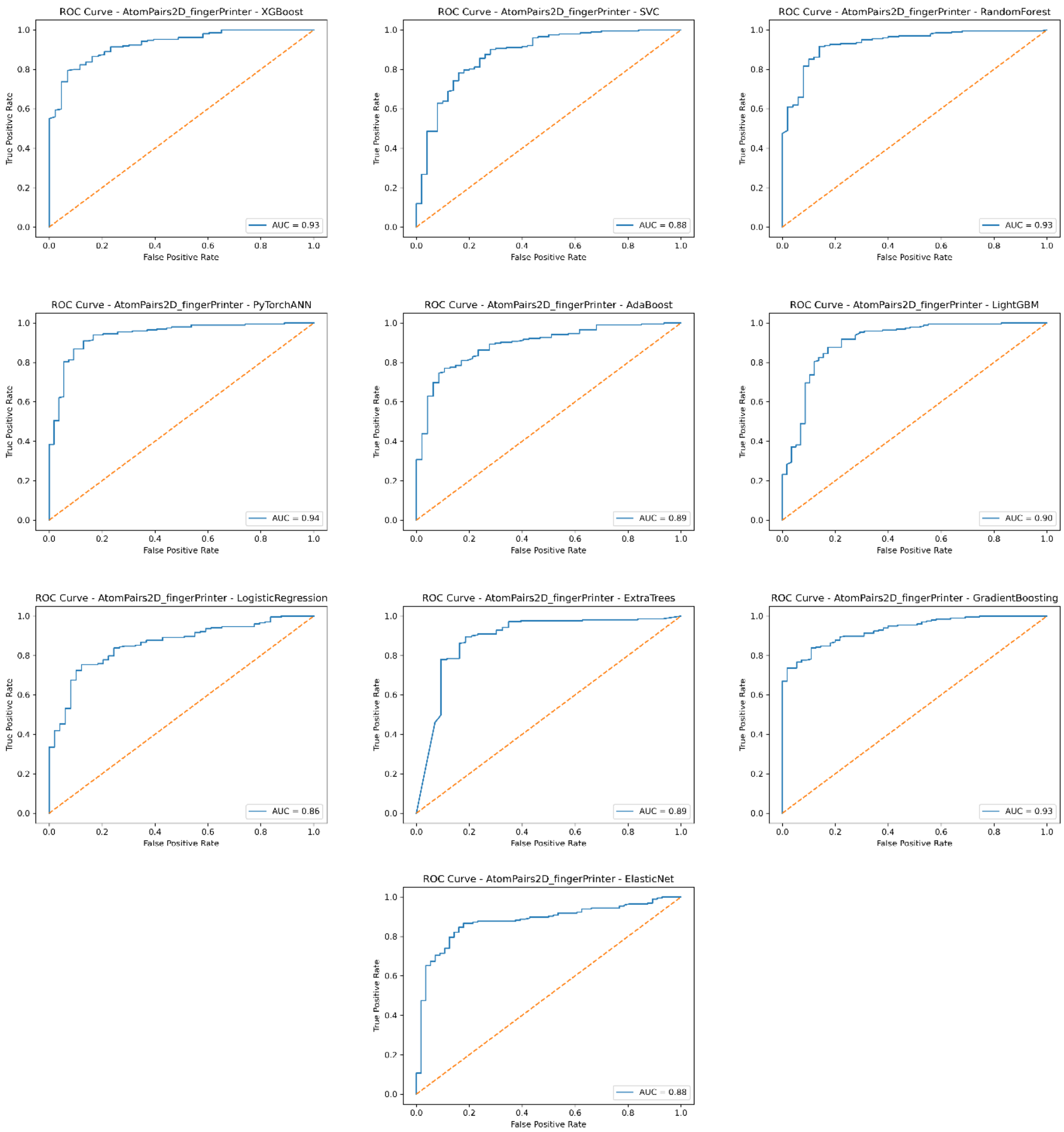

Figure S4. ROC curves for AtomPairs2D_FingerPrinter descriptor across eleven classifiers. Top-performing models included XGBoost (AUC = 0.93) and PyTorchANN (AUC = 0.94), indicating high discriminative ability.

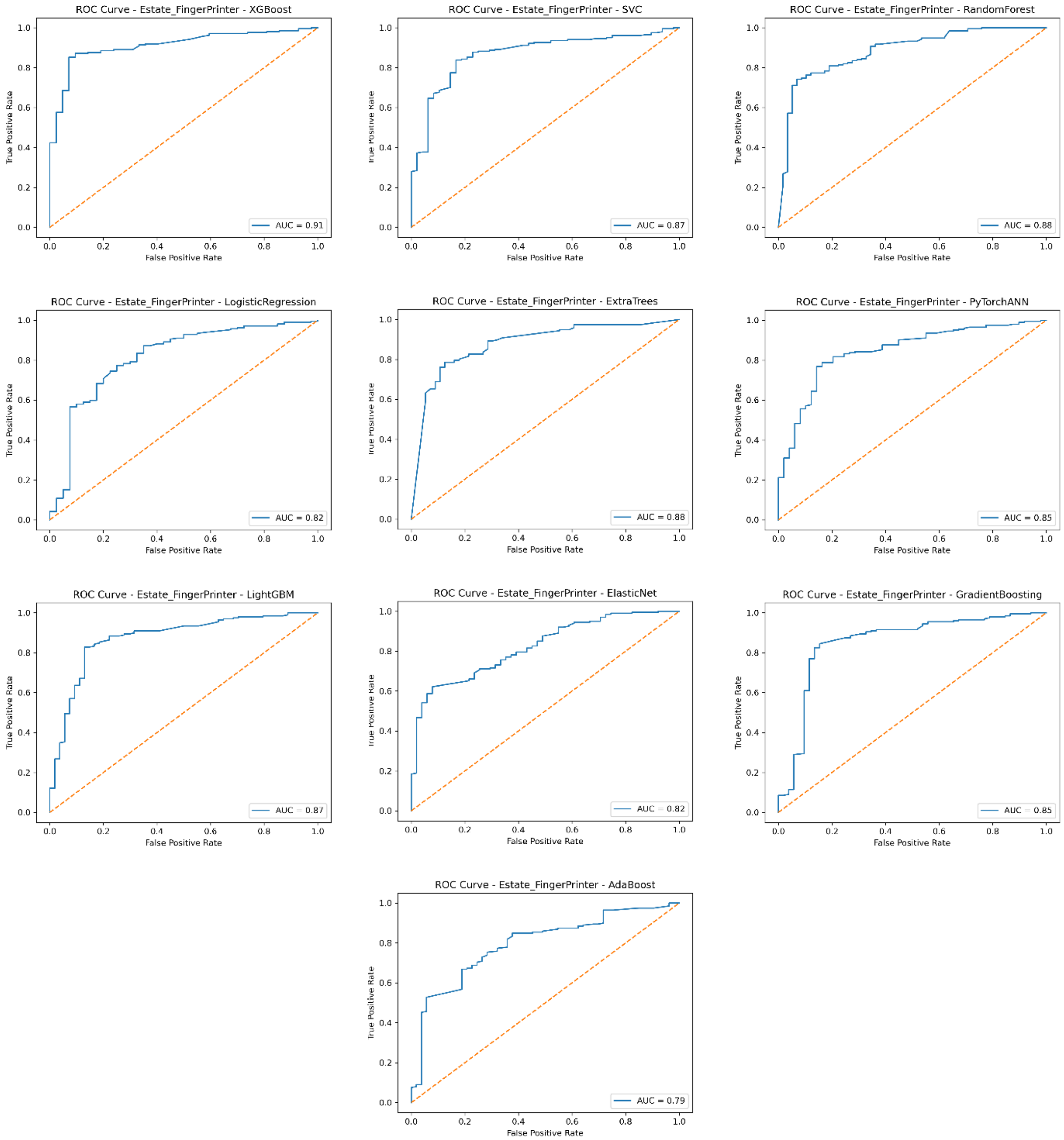

Figure S5. ROC curves for Estate_FingerPrinter models. GradientBoosting and XGBoost achieved AUC values of 0.85 and 0.91, respectively. AdaBoost performed comparatively lower (AUC = 0.79).

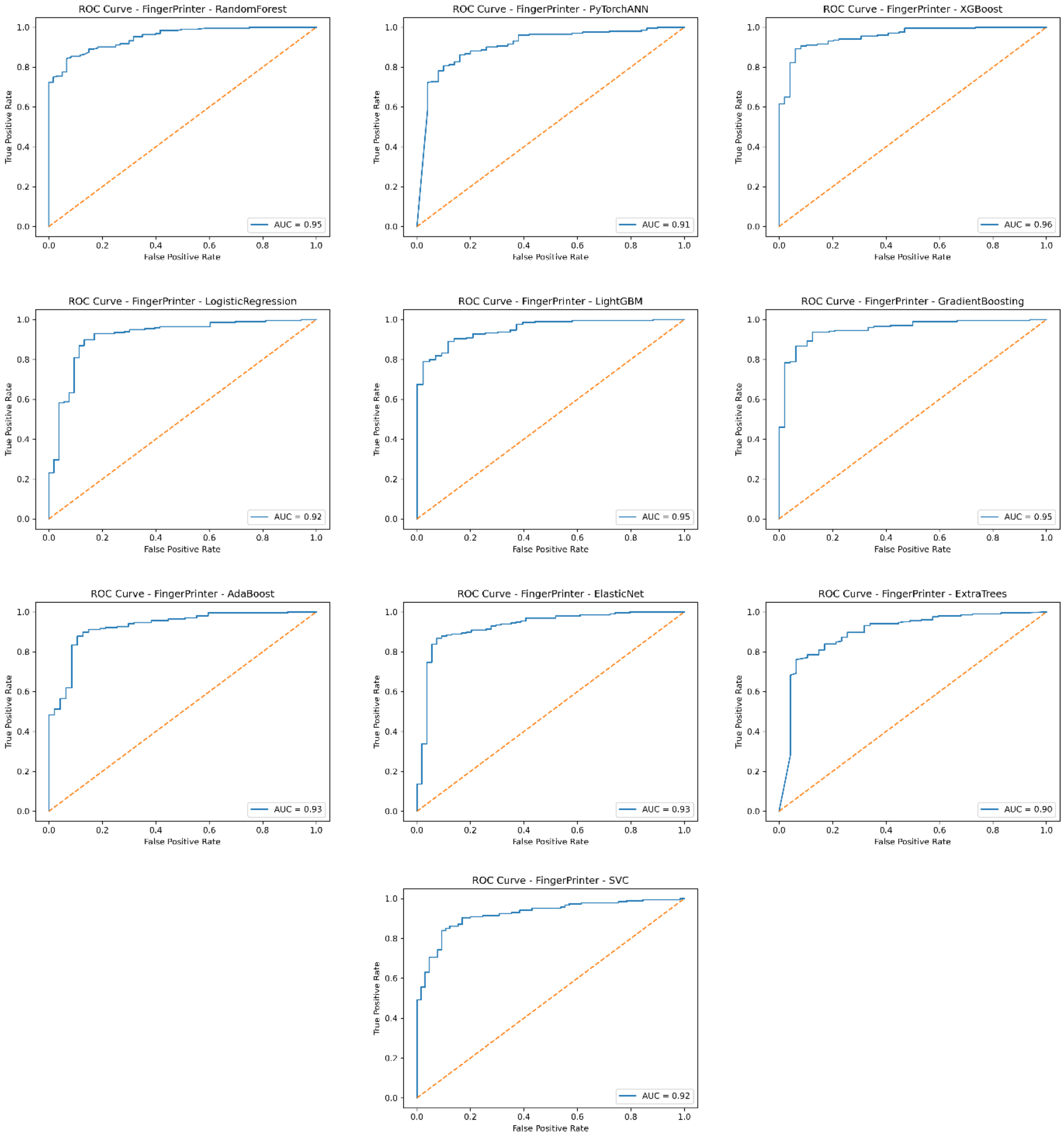

Figure S6. ROC curves for the generic FingerPrinter descriptor across all classifiers. LightGBM, XGBoost, and GradientBoosting yielded the highest AUCs (~0.95), confirming robust predictive performance.

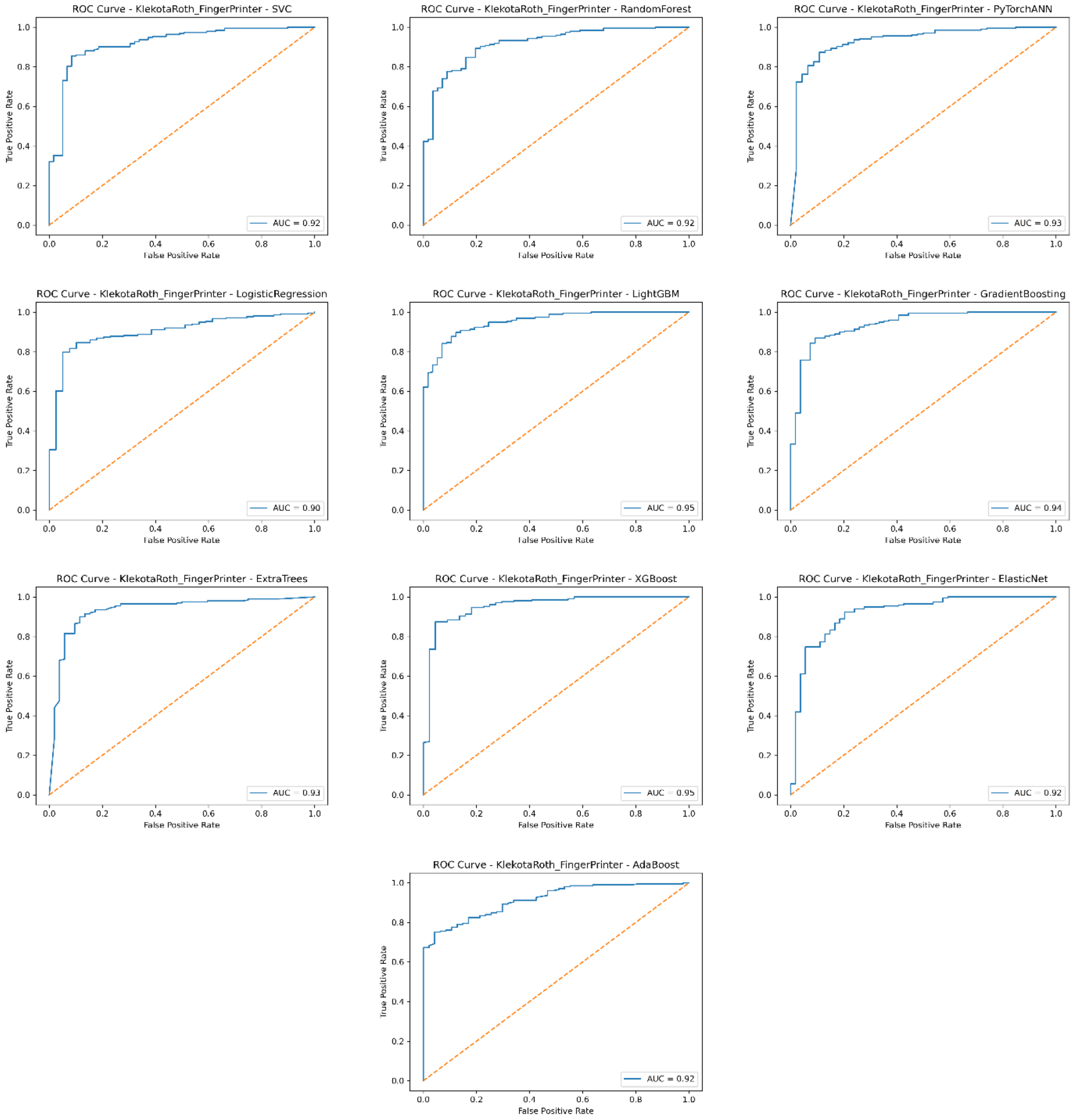

Figure S7. ROC curves for models using the KlekotaRoth_FingerPrinter descriptor. XGBoost and LightGBM provided the highest classification accuracy, each with AUCs of 0.95.

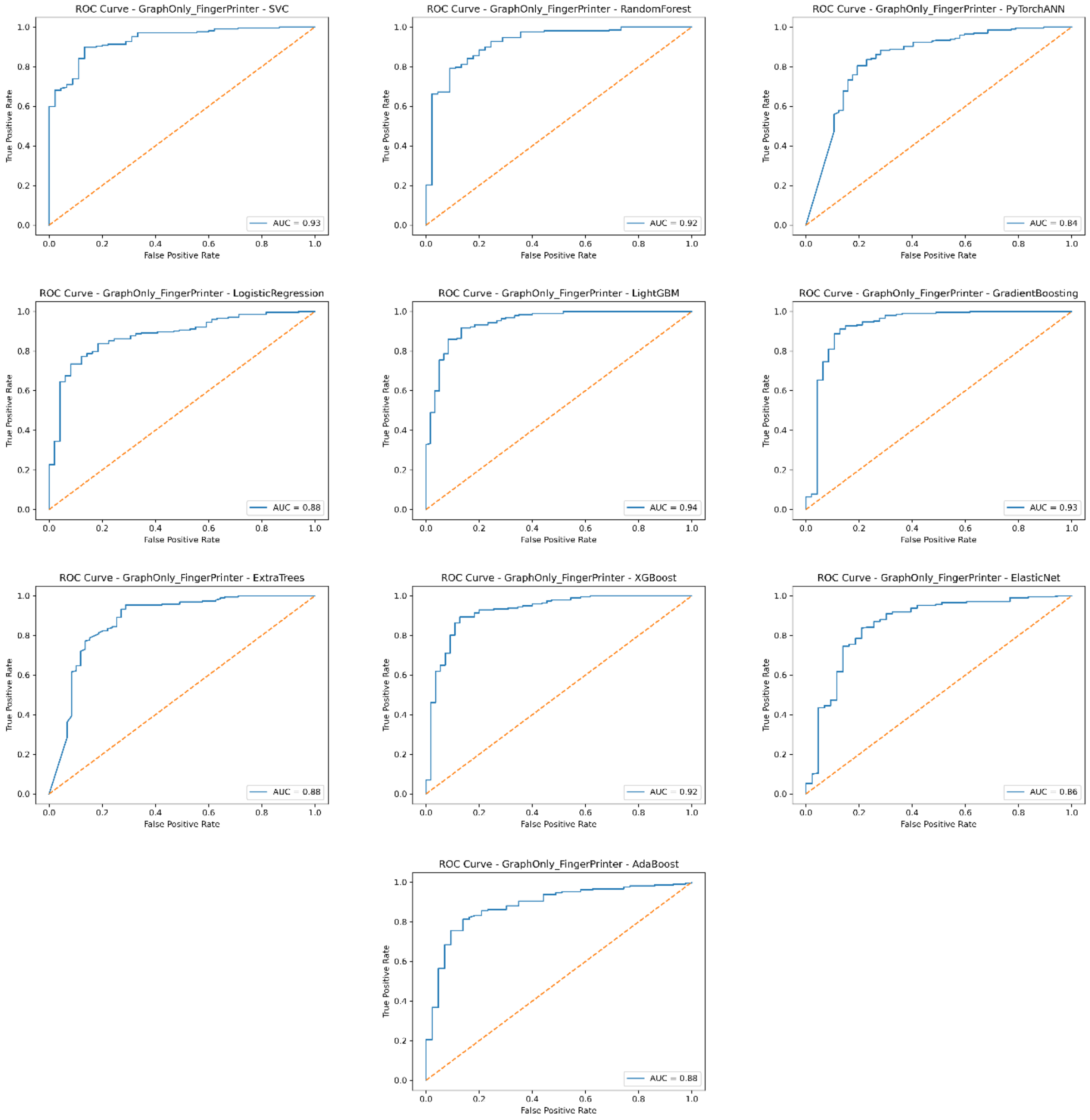

Figure S8. ROC curves for GraphOnly_FingerPrinter models. LightGBM and XGBoost performed consistently well (AUC = 0.94 and 0.92), with SVC also achieving high accuracy (AUC = 0.92).

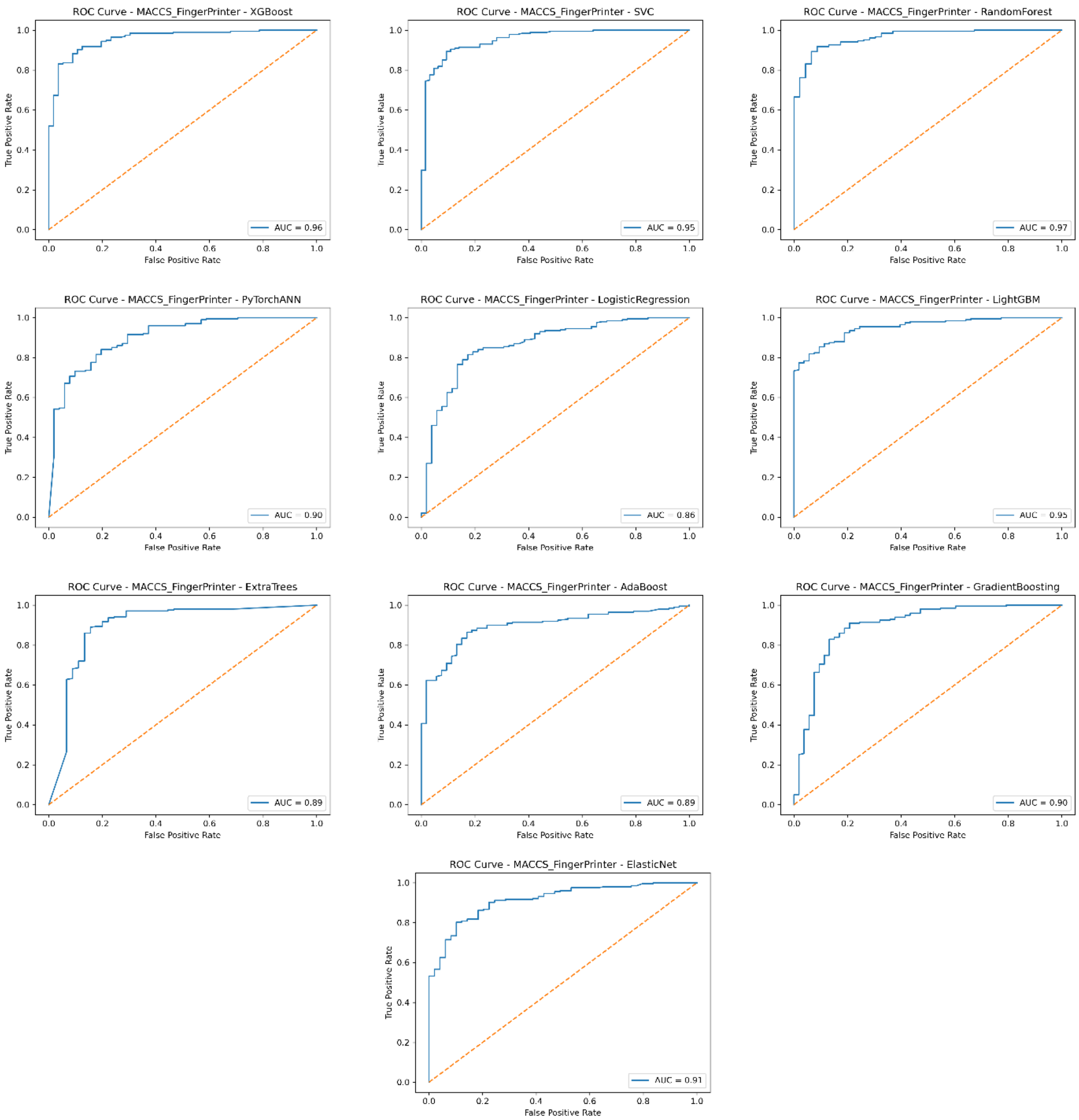

Figure S9. ROC curves for MACCS_FingerPrinter models. Performance was highest for XGBoost (AUC = 0.96), followed closely by LightGBM and GradientBoosting (AUC = 0.95 and 0.90, respectively).

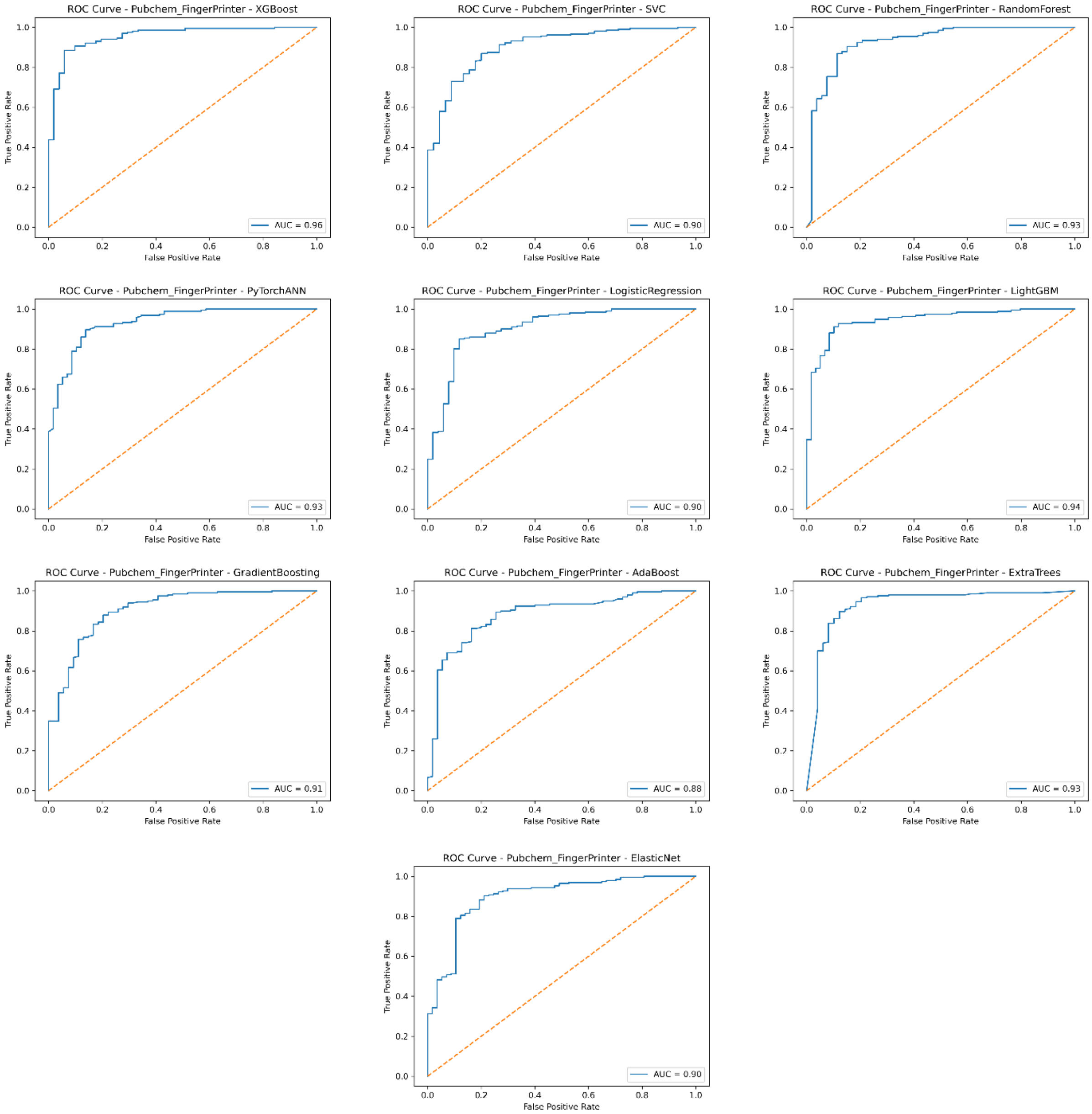

Figure S10. ROC curves for PubChem_FingerPrinter-based models. Most classifiers achieved AUC > 0.90, with the highest observed for XGBoost (AUC = 0.96).

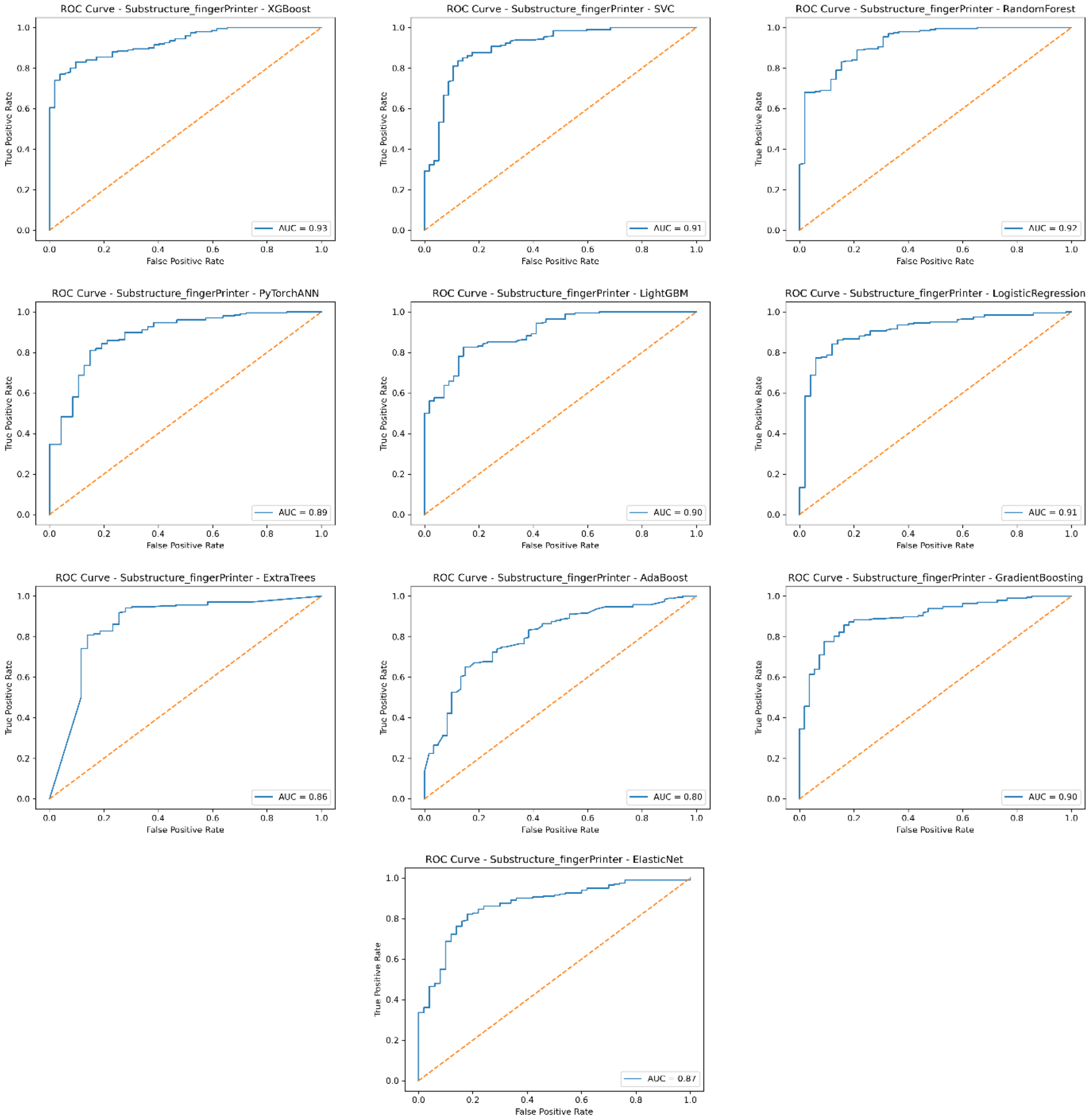

Figure S11. ROC curves for Substructure_FingerPrinter models. XGBoost and LightGBM showed AUC values of 0.93 and 0.90, respectively, outperforming other classifiers.

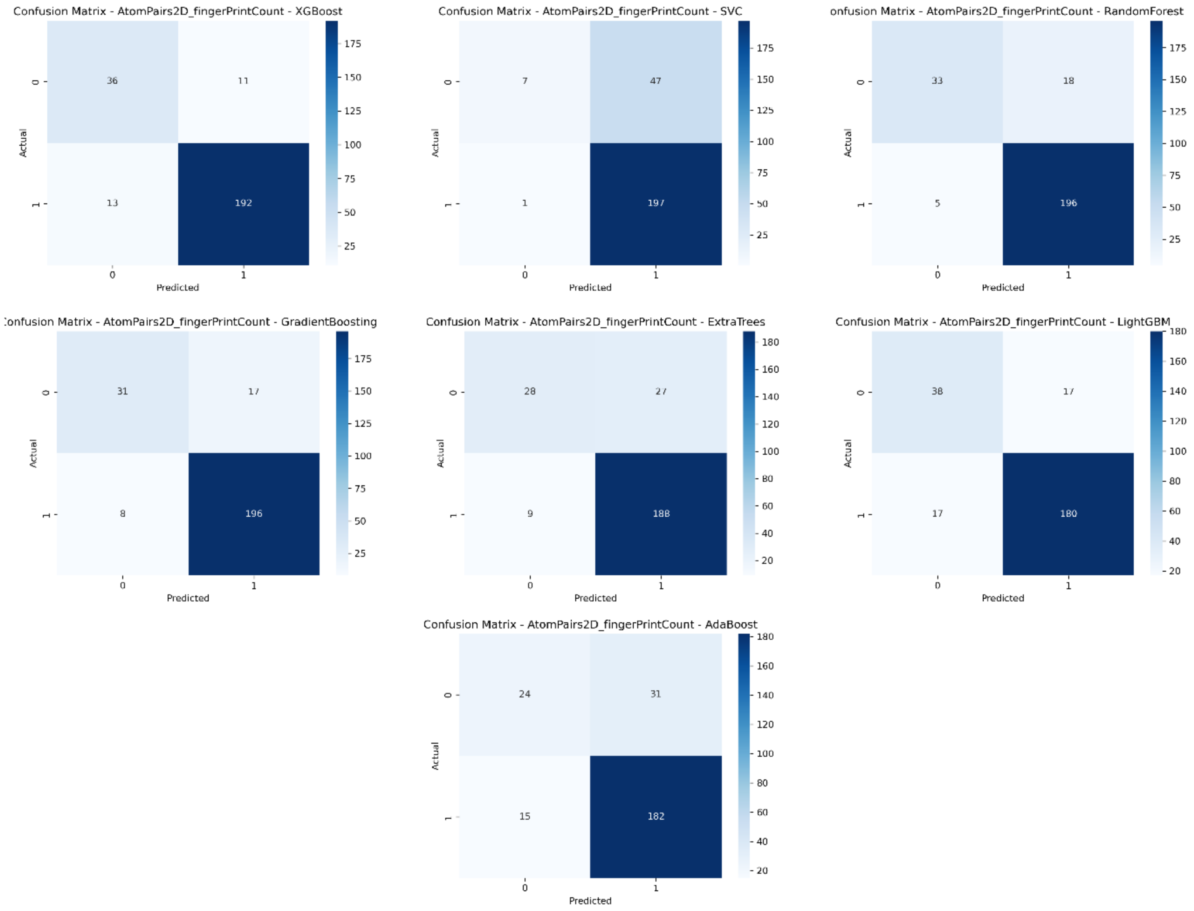

Figure S12. Confusion matrices for AtomPairs2D_fingerPrintCount across different machine learning algorithms.

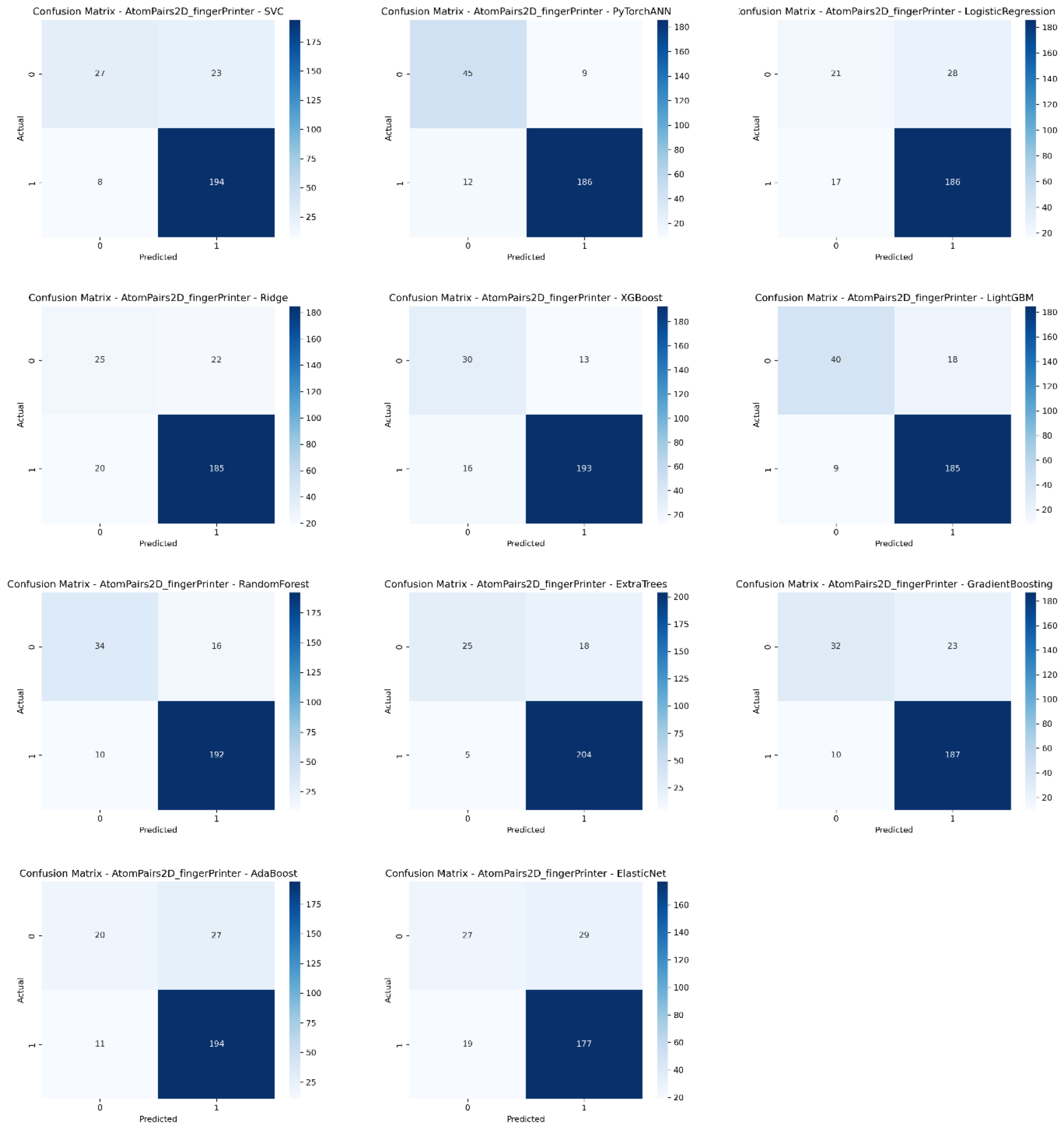

Figure S13. Confusion matrices for AtomPairs2D_FingerPrinter descriptors. Models were evaluated on their ability to accurately classify compounds using 2D atom pair fingerprints.

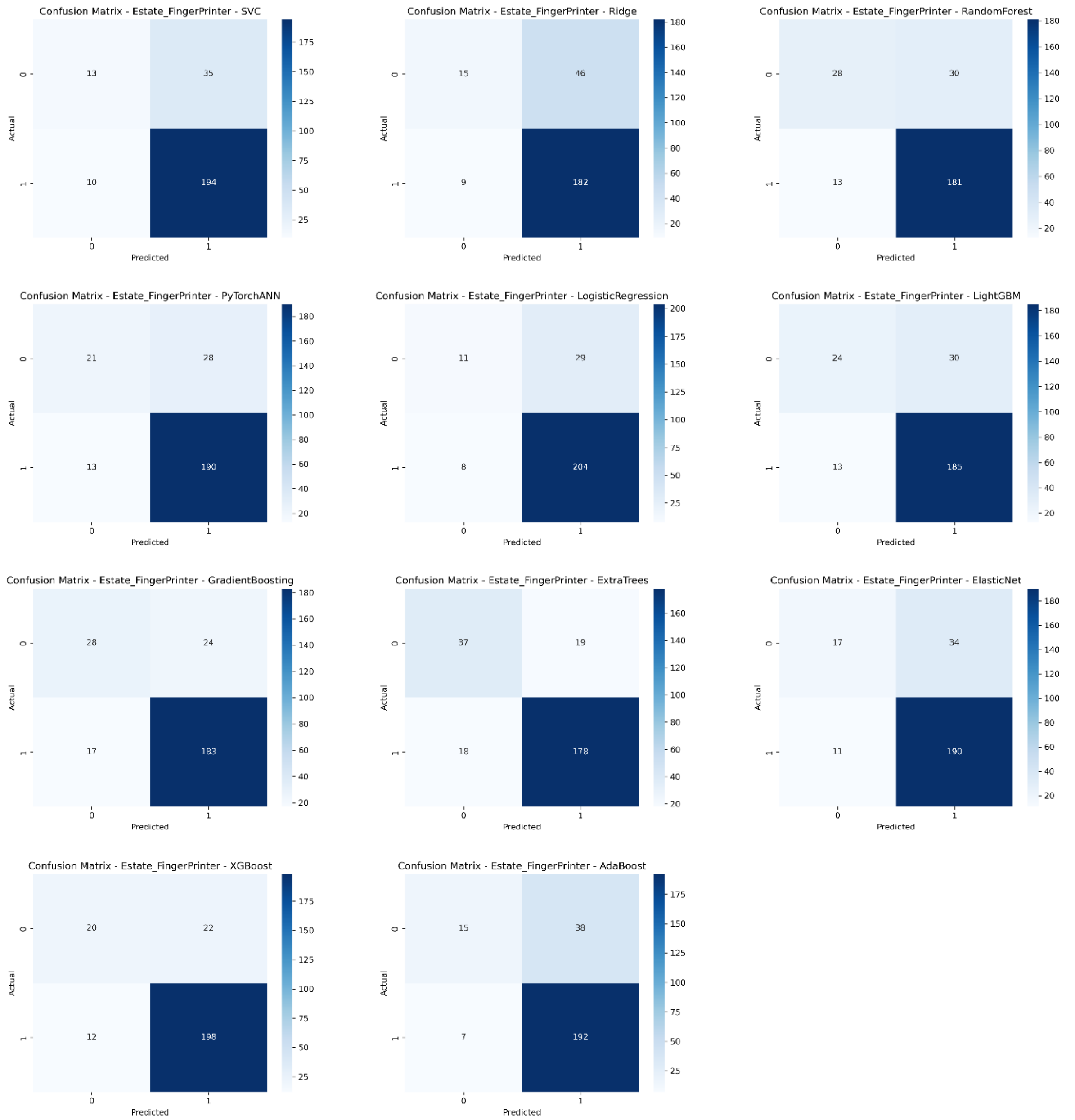

Figure S14. Confusion matrices for the Estate FingerPrinter descriptor across all tested models. Estate fingerprints provided varying resolution of bioactivity patterns as reflected in classification outcomes.

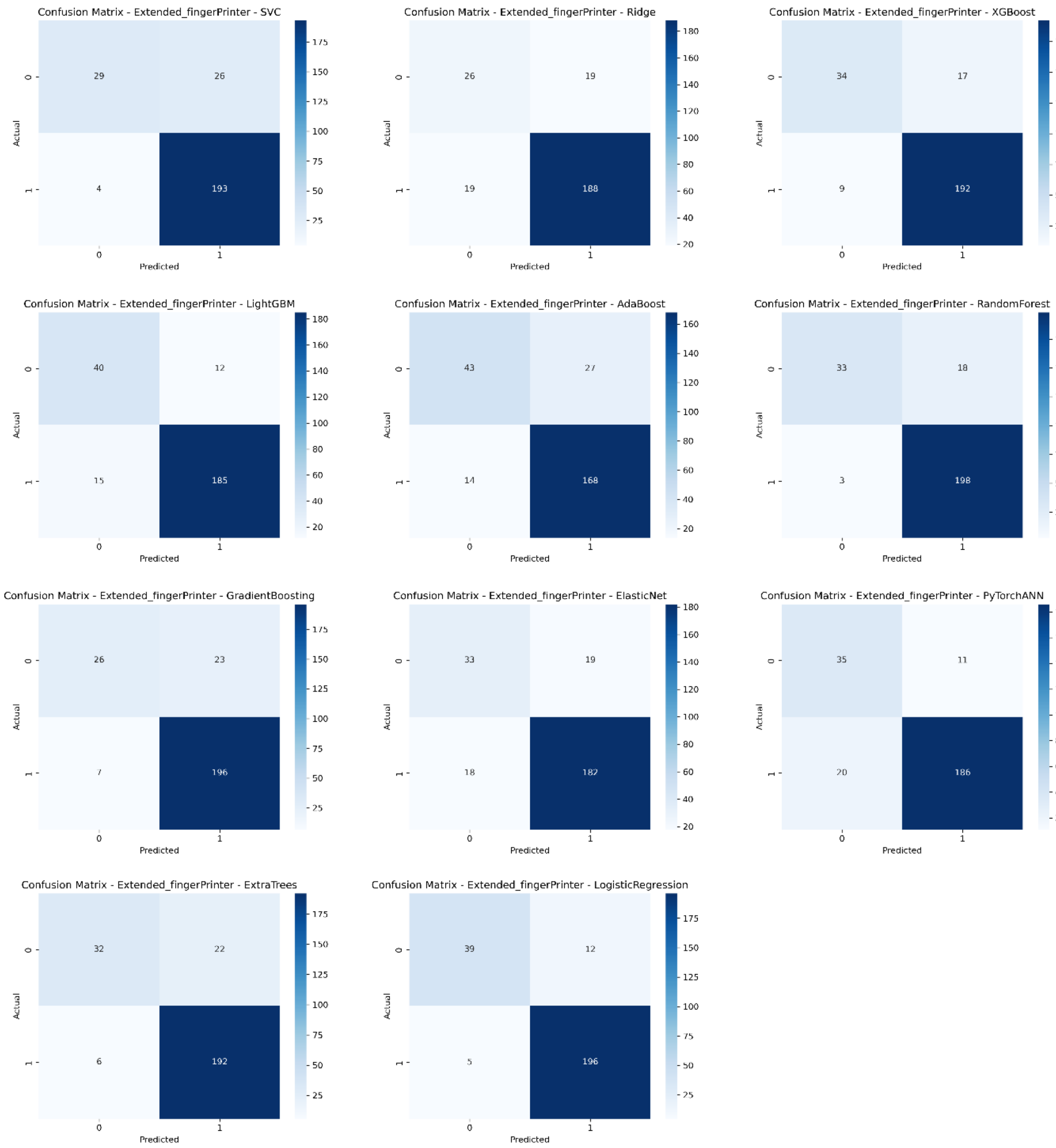

Figure S15. Confusion matrices for the Extended FingerPrinter descriptor with 10 machine learning classifiers. Performance varied across models, indicating model-dependent effectiveness of the extended fingerprint features.

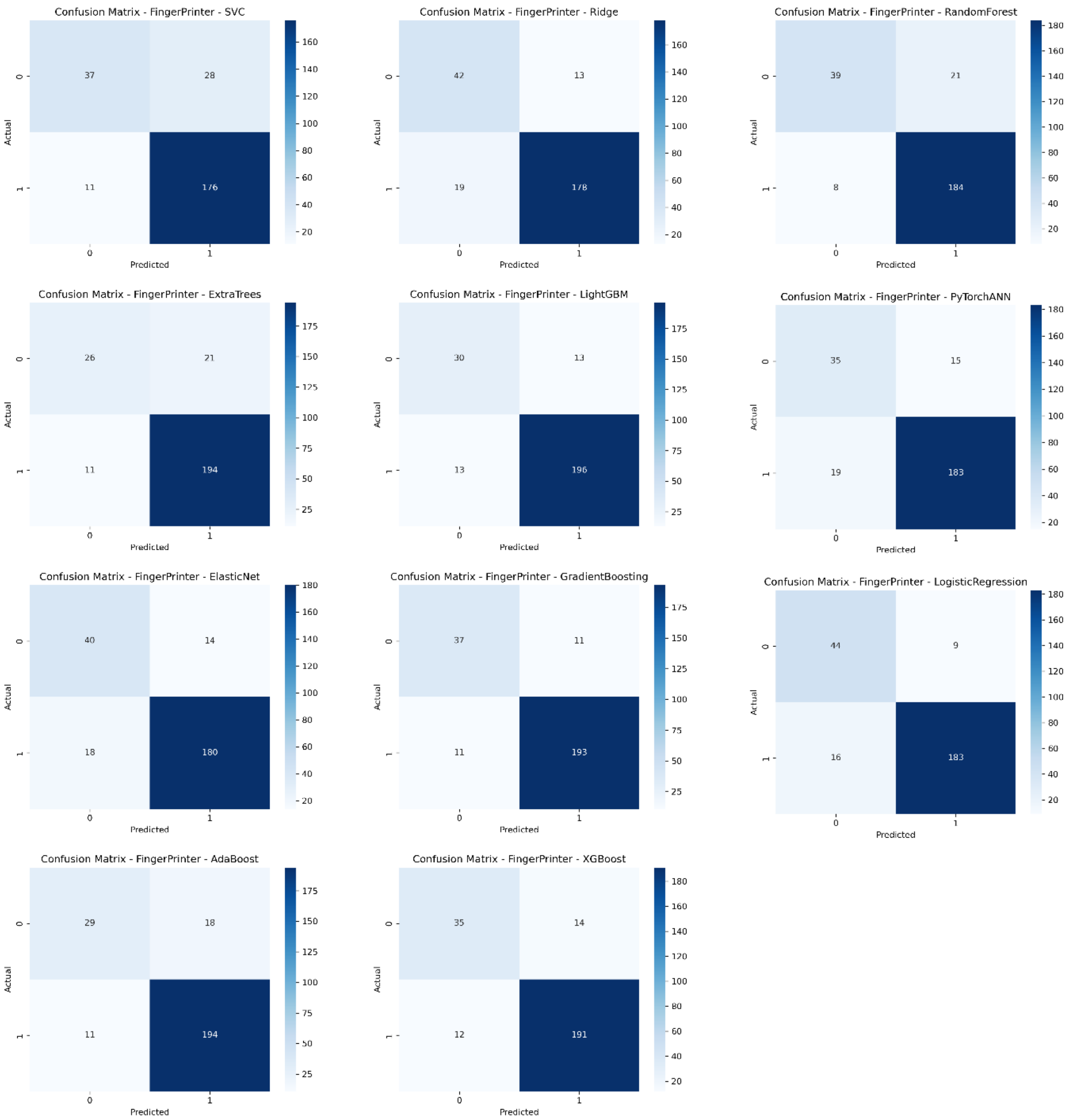

Figure S16. Confusion matrices for the standard FingerPrinter descriptor. These results demonstrate the predictive consistency of this classical representation across most models.

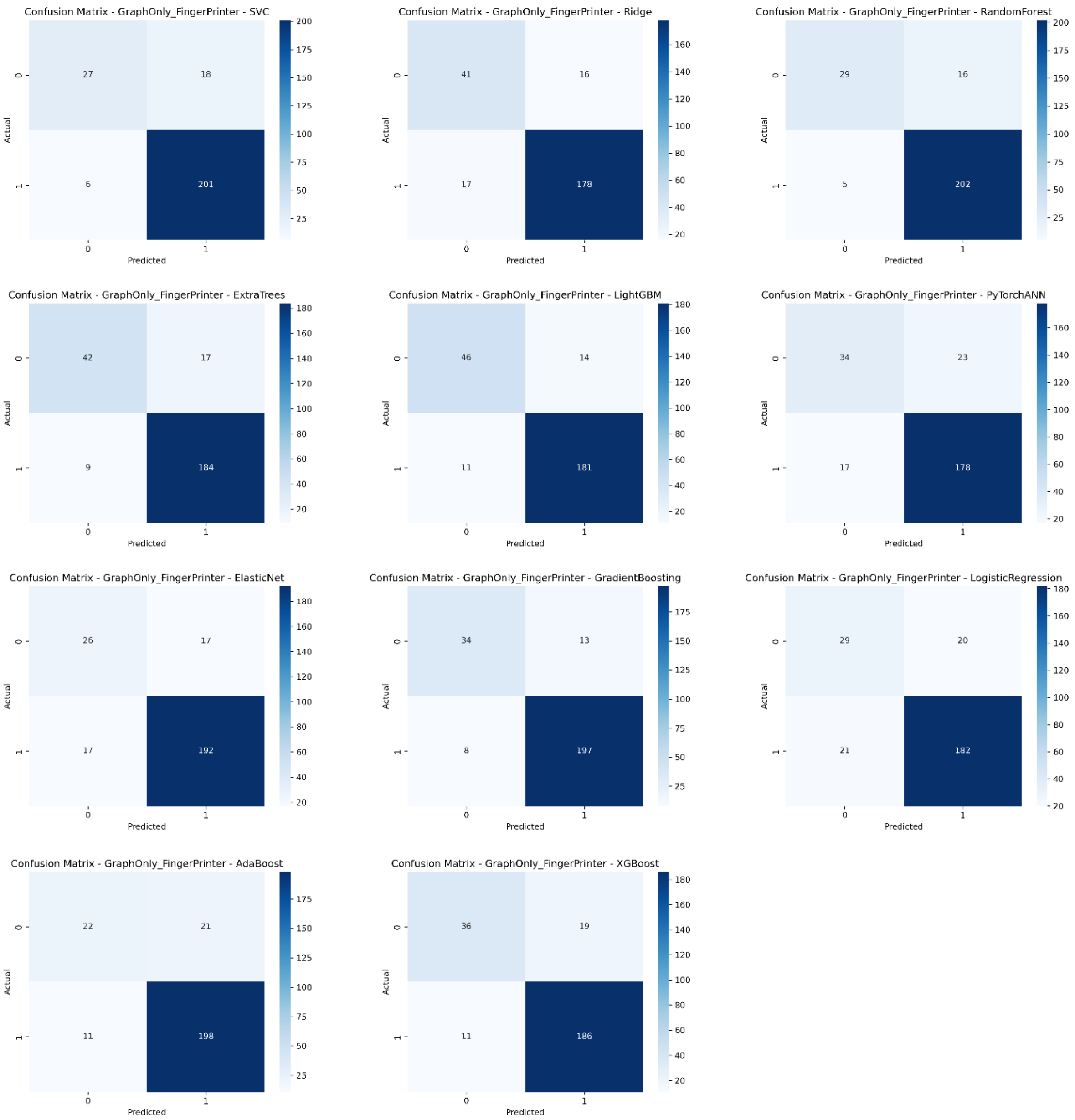

Figure S17. Confusion matrices for GraphOnly_FingerPrinter representations. Classification patterns reflect the models' handling of topological graph-based features in bioactivity prediction.

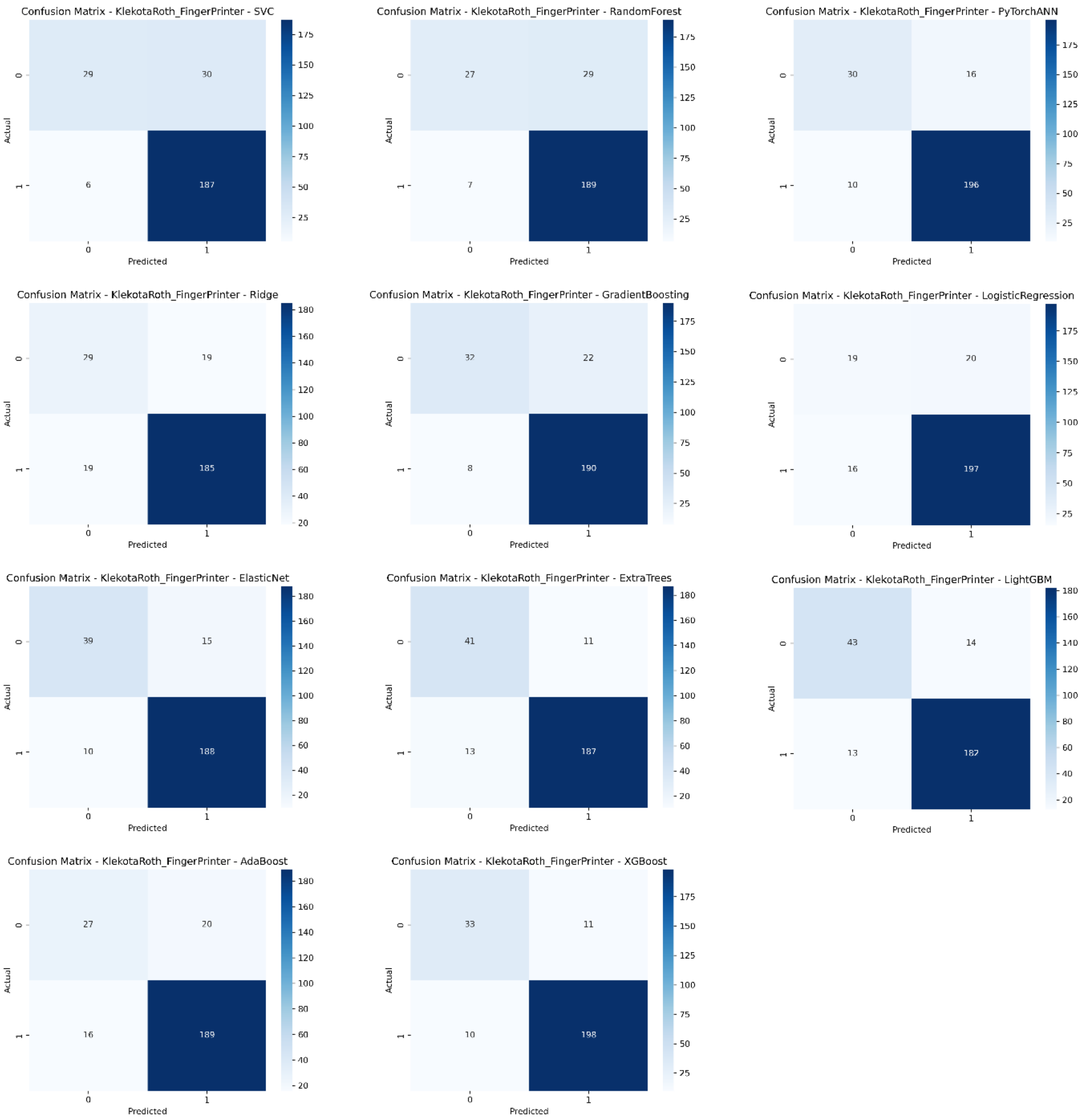

Figure S18. Confusion matrices for KlekotaRoth_FingerPrinter, showing strong predictive separability in some models and modest confusion in others, underlining the impact of feature dimensionality.

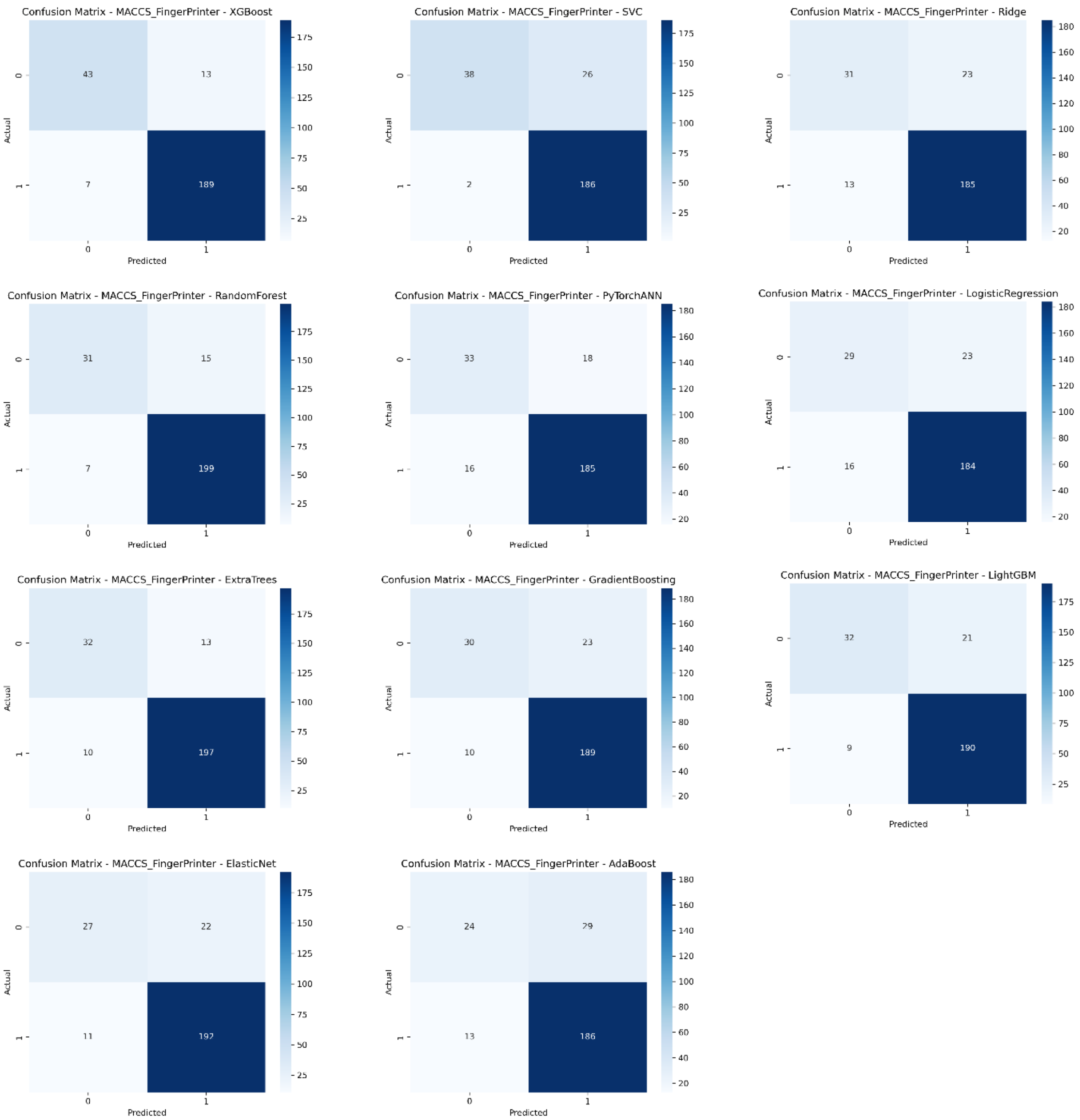

Figure S19. Confusion matrices for MACCS_FingerPrinter across all classifiers. MACCS keys provide compact yet informative features for bioactivity classification.

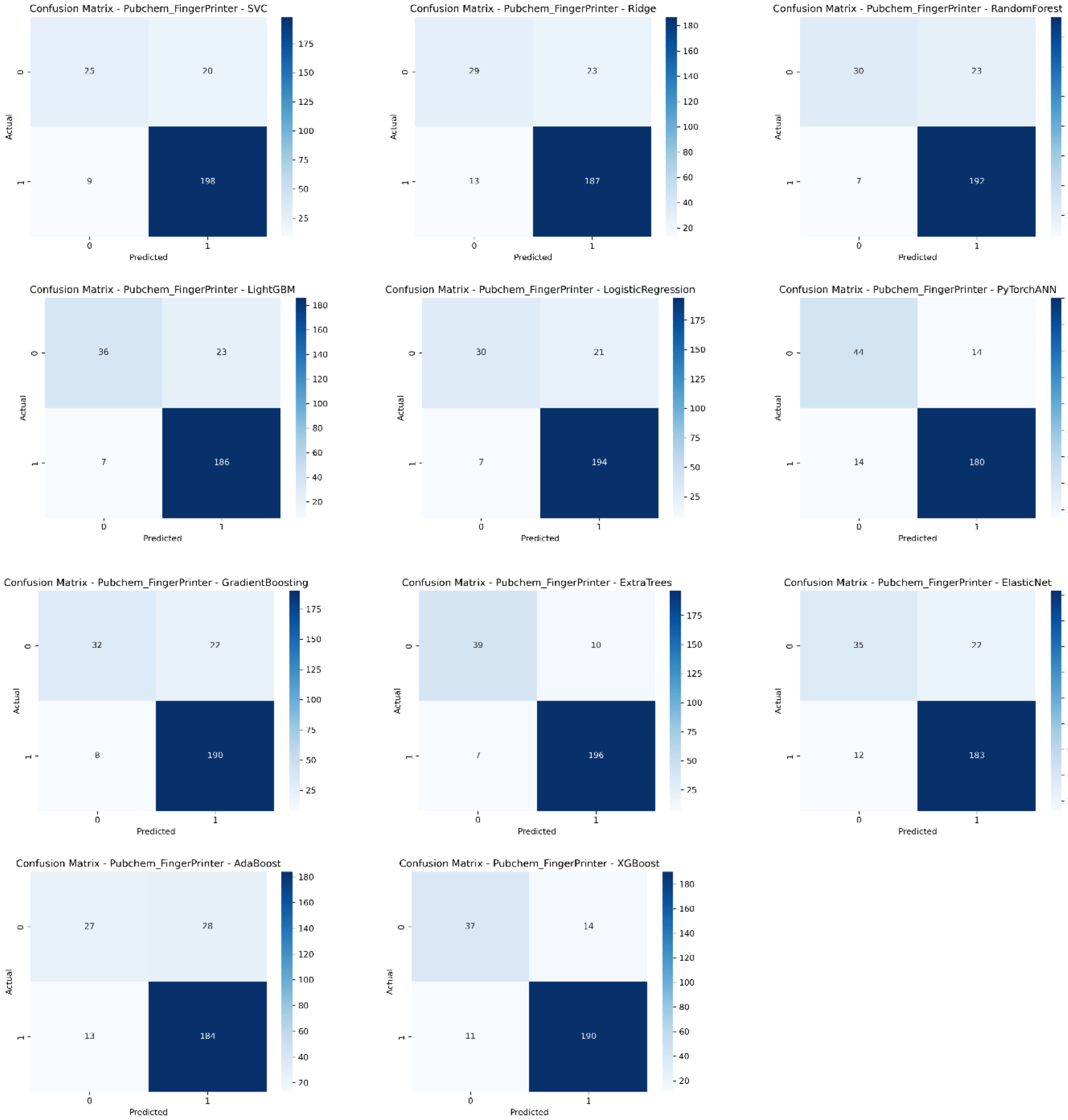

Figure S20. Confusion matrices for Pubchem_FingerPrinter across 10 models. The results reflect how standardized structural keys perform across diverse machine learning settings.

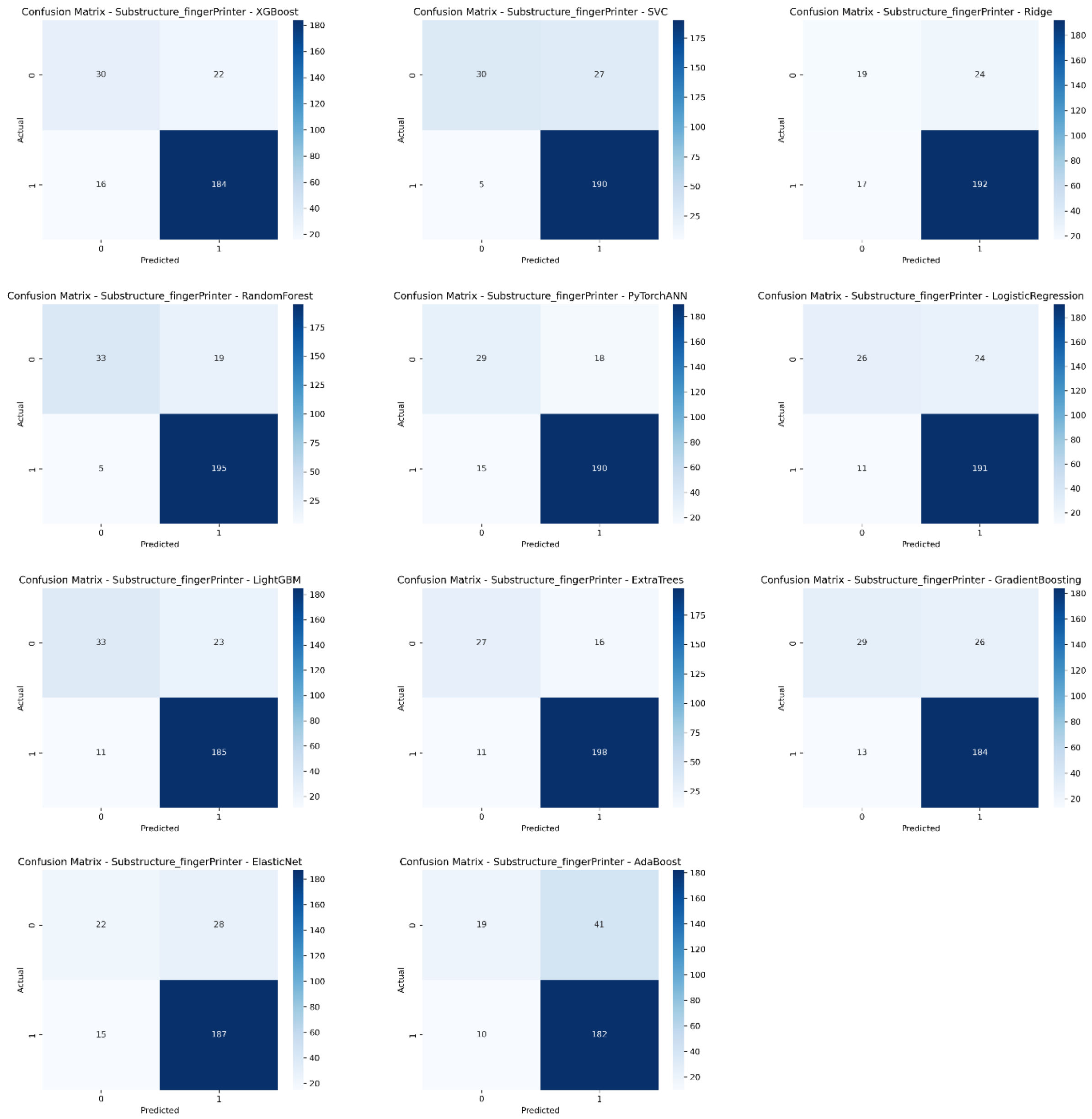

Figure S21. Confusion matrices for classification using the Substructure FingerPrinter descriptor across 10 machine learning models.

Table S1. Comprehensive evaluation of 10 machine learning algorithms across 10 molecular fingerprint descriptors for bioactivity prediction. Performance metrics include Accuracy, Area Under the Receiver Operating Characteristic Curve (AUC), F1 score, Recall, Specificity, and Precision. Models are ranked by the average of AUC and Accuracy (AUC_Acc_Avg), used as a balanced metric for overall performance.

Descriptor	Model	Accuracy	AUC	F1	Recall	Specificity	Precision	AUC_Acc_Avg
FingerPrinter	LightGBM	0.9	0.826	0.938	0.952	0.699	0.924	0.863
Extended_fingerPrinter	LightGBM	0.902	0.823	0.939	0.955	0.692	0.924	0.8625
FingerPrinter	XGBoost	0.899	0.825	0.937	0.951	0.7	0.924	0.862
Extended_fingerPrinter	XGBoost	0.899	0.825	0.937	0.952	0.697	0.923	0.862
AtomPairs2D_fingerPrinter	LightGBM	0.895	0.826	0.934	0.944	0.708	0.925	0.8605
FingerPrinter	ElasticNet	0.895	0.826	0.935	0.945	0.707	0.925	0.8605
AtomPairs2D_fingerPrinter	XGBoost	0.894	0.824	0.934	0.942	0.707	0.927	0.859
FingerPrinter	LogisticRegression	0.893	0.825	0.933	0.943	0.707	0.924	0.859
Extended_fingerPrinter	ExtraTrees	0.9	0.817	0.939	0.959	0.676	0.919	0.8585
MACCS_FingerPrinter	XGBoost	0.893	0.822	0.933	0.942	0.702	0.926	0.8575
KlekotaRoth_FingerPrinter	XGBoost	0.893	0.822	0.934	0.943	0.701	0.924	0.8575
AtomPairs2D_fingerPrinter	ExtraTrees	0.898	0.817	0.937	0.956	0.678	0.919	0.8575
MACCS_FingerPrinter	LightGBM	0.895	0.819	0.935	0.949	0.689	0.921	0.857
FingerPrinter	GradientBoosting	0.895	0.818	0.935	0.947	0.689	0.923	0.8565
KlekotaRoth_FingerPrinter	LightGBM	0.89	0.823	0.931	0.938	0.707	0.925	0.8565
Extended_fingerPrinter	GradientBoosting	0.894	0.817	0.934	0.949	0.684	0.919	0.8555
Extended_fingerPrinter	RandomForest	0.898	0.811	0.937	0.96	0.661	0.916	0.8545
FingerPrinter	PyTorchANN	0.887	0.821	0.929	0.934	0.709	0.925	0.854
Extended_fingerPrinter	PyTorchANN	0.888	0.819	0.929	0.938	0.701	0.922	0.8535
Extended_fingerPrinter	LogisticRegression	0.891	0.816	0.932	0.943	0.689	0.922	0.8535
FingerPrinter	ExtraTrees	0.896	0.81	0.936	0.959	0.662	0.915	0.853
Extended_fingerPrinter	ElasticNet	0.889	0.816	0.931	0.94	0.692	0.922	0.8525
Pubchem_FingerPrinter	ExtraTrees	0.892	0.813	0.933	0.948	0.678	0.919	0.8525
AtomPairs2D_fingerPrinter	RandomForest	0.898	0.805	0.938	0.962	0.649	0.915	0.8515

FingerPrinter	AdaBoost	0.887	0.813	0.93	0.94	0.686	0.919	0.85
KlekotaRoth_FingerPrinter	ExtraTrees	0.892	0.805	0.934	0.953	0.657	0.915	0.8485
FingerPrinter	RandomForest	0.895	0.801	0.935	0.963	0.638	0.91	0.848
Pubchem_FingerPrinter	XGBoost	0.885	0.811	0.928	0.937	0.685	0.919	0.848
MACCS_FingerPrinter	ExtraTrees	0.888	0.807	0.93	0.945	0.669	0.916	0.8475
Pubchem_FingerPrinter	LightGBM	0.886	0.807	0.929	0.939	0.675	0.92	0.8465
Pubchem_FingerPrinter	RandomForest	0.89	0.801	0.932	0.953	0.65	0.912	0.8455
MACCS_FingerPrinter	RandomForest	0.89	0.8	0.932	0.954	0.647	0.912	0.845
MACCS_FingerPrinter	GradientBoosting	0.887	0.801	0.93	0.947	0.655	0.915	0.844
MACCS_FingerPrinter	PyTorchANN	0.88	0.804	0.925	0.934	0.675	0.917	0.842
AtomPairs2D_fingerPrinter	PyTorchANN	0.879	0.805	0.924	0.929	0.681	0.92	0.842
MACCS_FingerPrinter	SVC	0.887	0.796	0.93	0.951	0.641	0.911	0.8415
GraphOnly_FingerPrinter	LightGBM	0.883	0.799	0.927	0.943	0.654	0.912	0.841
Pubchem_FingerPrinter	GradientBoosting	0.884	0.798	0.928	0.944	0.651	0.913	0.841
Extended_fingerPrinter	SVC	0.887	0.79	0.931	0.958	0.622	0.906	0.8385
Extended_fingerPrinter	AdaBoost	0.88	0.795	0.925	0.941	0.649	0.91	0.8375
GraphOnly_FingerPrinter	ExtraTrees	0.886	0.787	0.93	0.953	0.622	0.908	0.8365
Substructure_fingerPrinter	LightGBM	0.878	0.795	0.924	0.936	0.655	0.912	0.8365
GraphOnly_FingerPrinter	XGBoost	0.879	0.794	0.924	0.94	0.648	0.909	0.8365
Substructure_fingerPrinter	ExtraTrees	0.877	0.795	0.923	0.936	0.654	0.911	0.836
Pubchem_FingerPrinter	PyTorchANN	0.876	0.796	0.922	0.93	0.661	0.915	0.836
Pubchem_FingerPrinter	ElasticNet	0.88	0.791	0.926	0.942	0.64	0.91	0.8355
KlekotaRoth_FingerPrinter	Ridge	0.876	0.794	0.923	0.932	0.656	0.914	0.835
FingerPrinter	SVC	0.884	0.785	0.929	0.953	0.617	0.906	0.8345
KlekotaRoth_FingerPrinter	PyTorchANN	0.871	0.797	0.919	0.924	0.669	0.915	0.834
KlekotaRoth_FingerPrinter	LogisticRegression	0.878	0.788	0.925	0.942	0.634	0.909	0.833
GraphOnly_FingerPrinter	RandomForest	0.886	0.78	0.93	0.959	0.601	0.904	0.833

Substructure_fingerPrinter	XGBoost	0.874	0.792	0.921	0.931	0.654	0.912	0.833
AtomPairs2D_fingerPrinter	GradientBoosting	0.875	0.789	0.922	0.936	0.641	0.909	0.832
KlekotaRoth_FingerPrinter	RandomForest	0.889	0.775	0.933	0.969	0.58	0.899	0.832
KlekotaRoth_FingerPrinter	ElasticNet	0.876	0.785	0.923	0.942	0.628	0.905	0.8305
GraphOnly_FingerPrinter	Ridge	0.865	0.791	0.915	0.918	0.665	0.913	0.828
Pubchem_FingerPrinter	LogisticRegression	0.876	0.78	0.923	0.942	0.618	0.906	0.828
GraphOnly_FingerPrinter	PyTorchANN	0.869	0.785	0.918	0.927	0.642	0.91	0.827
Substructure_fingerPrinter	PyTorchANN	0.868	0.785	0.918	0.929	0.641	0.907	0.8265
FingerPrinter	Ridge	0.858	0.795	0.91	0.903	0.686	0.917	0.8265
Substructure_fingerPrinter	RandomForest	0.876	0.776	0.924	0.948	0.604	0.901	0.826
GraphOnly_FingerPrinter	LogisticRegression	0.868	0.779	0.918	0.93	0.629	0.906	0.8235
GraphOnly_FingerPrinter	ElasticNet	0.867	0.774	0.917	0.933	0.616	0.902	0.8205
Extended_fingerPrinter	Ridge	0.849	0.791	0.903	0.889	0.692	0.919	0.82
GraphOnly_FingerPrinter	GradientBoosting	0.87	0.769	0.919	0.942	0.597	0.899	0.8195
Pubchem_FingerPrinter	Ridge	0.867	0.772	0.917	0.934	0.609	0.901	0.8195
KlekotaRoth_FingerPrinter	GradientBoosting	0.874	0.761	0.923	0.954	0.569	0.894	0.8175
MACCS_FingerPrinter	LogisticRegression	0.865	0.769	0.916	0.933	0.606	0.901	0.817
GraphOnly_FingerPrinter	SVC	0.874	0.759	0.923	0.956	0.563	0.893	0.8165
MACCS_FingerPrinter	ElasticNet	0.863	0.77	0.915	0.93	0.61	0.9	0.8165
Pubchem_FingerPrinter	SVC	0.876	0.755	0.925	0.962	0.547	0.891	0.8155
MACCS_FingerPrinter	Ridge	0.862	0.764	0.914	0.93	0.599	0.899	0.813
Substructure_fingerPrinter	GradientBoosting	0.863	0.758	0.916	0.939	0.577	0.894	0.8105
AtomPairs2D_fingerPrinter	SVC	0.867	0.748	0.919	0.954	0.543	0.887	0.8075
KlekotaRoth_FingerPrinter	SVC	0.872	0.736	0.923	0.969	0.504	0.882	0.804
Pubchem_FingerPrinter	AdaBoost	0.858	0.737	0.913	0.946	0.528	0.883	0.7975
Estate_FingerPrinter	ExtraTrees	0.838	0.751	0.898	0.899	0.603	0.897	0.7945
Substructure_fingerPrinter	SVC	0.867	0.713	0.921	0.975	0.45	0.873	0.79

KlekotaRoth_FingerPrinter	AdaBoost	0.855	0.724	0.912	0.947	0.501	0.88	0.7895
Estate_FingerPrinter	RandomForest	0.84	0.732	0.9	0.918	0.545	0.884	0.786
Estate_FingerPrinter	XGBoost	0.838	0.725	0.9	0.917	0.533	0.884	0.7815
Estate_FingerPrinter	PyTorchANN	0.832	0.728	0.895	0.905	0.551	0.887	0.78
AtomPairs2D_fingerPrinter	LogisticRegression	0.84	0.715	0.902	0.927	0.504	0.878	0.7775
Estate_FingerPrinter	LightGBM	0.837	0.716	0.899	0.92	0.512	0.88	0.7765
Substructure_fingerPrinter	LogisticRegression	0.845	0.707	0.906	0.943	0.472	0.873	0.776
MACCS_FingerPrinter	AdaBoost	0.84	0.711	0.902	0.933	0.489	0.874	0.7755
Substructure_fingerPrinter	ElasticNet	0.844	0.703	0.905	0.943	0.462	0.871	0.7735
AtomPairs2D_fingerPrinter	Ridge	0.835	0.706	0.899	0.925	0.487	0.875	0.7705
AtomPairs2D_fingerPrinter	ElasticNet	0.832	0.707	0.897	0.925	0.488	0.871	0.7695
Substructure_fingerPrinter	Ridge	0.845	0.693	0.907	0.952	0.433	0.866	0.769
GraphOnly_FingerPrinter	AdaBoost	0.837	0.699	0.901	0.939	0.46	0.866	0.768
Estate_FingerPrinter	GradientBoosting	0.837	0.699	0.901	0.933	0.464	0.871	0.768
AtomPairs2D_fingerPrinter	AdaBoost	0.833	0.687	0.899	0.942	0.432	0.86	0.76
Substructure_fingerPrinter	AdaBoost	0.84	0.679	0.905	0.952	0.407	0.862	0.7595
Estate_FingerPrinter	SVC	0.827	0.636	0.898	0.96	0.313	0.845	0.7315
Estate_FingerPrinter	ElasticNet	0.816	0.626	0.891	0.948	0.304	0.84	0.721
Estate_FingerPrinter	LogisticRegression	0.814	0.623	0.89	0.95	0.296	0.837	0.7185
Estate_FingerPrinter	Ridge	0.809	0.604	0.888	0.957	0.251	0.828	0.7065
Estate_FingerPrinter	AdaBoost	0.799	0.594	0.881	0.95	0.238	0.823	0.6965

Table S2. Molecular interactions of top-ranked compounds and the reference ligand (PHA-E429) within the ALK active site. Hydrogen bond donors (HBD), hydrogen bond acceptors (HBA), ionic contacts, and π-H interactions are listed along with their respective interaction distances and estimated binding energies.

**Ligand Atoms**	**Receptor Atoms**	**Residues**	**Interactions**	**Distance (Å)**	**Energy (kcal/mol)**	**Ligand Atoms**
2XBA (PHA-E429)	N20	OE1	GLU118	HBD	3.6	-1.2
	N5	O	MET107	HBD	2.97	-2.4
	N2	O	GLU105	HBD	2.65	-3.6
	N4	N	MET107	HBA	2.72	-4.3
	N20	OE1	GLU118	Ionic	3.6	-1.5
	6-ring	CB	LEU30	π-H	4.5	-0.8
ZINC3870414	O11	O	GLU105	HBD	3.55	-0.6
	O12	O	MET107	HBD	2.69	-1.8
	O19	O	HIS32	HBD	2.69	-3.6
	O21	OD1	ASP111	HBD	2.54	-3.2
	O31	O	ARG161	HBD	2.71	-3
	O32	OD1	ASP178	HBD	2.59	-1.6
	O33	OD1	ASP178	HBD	2.5	-3.3
ZINC31155769	C	OD1	ASP111	HBD	3.25	-0.6
	O	OD1	ASP178	HBD	2.91	-1.8
	O	O	LEU30	HBD	3.07	-1.5
	O	N	ASP111	HBA	3.02	-2.3
	6-ring	CG1	VAL38	π-H	4.18	-0.6
ZINC15657732	O20	O	MET107	HBD	2.7	-3.7
	O28	O	ARG161	HBD	2.68	-3.6
	O30	OD1	ASP178	HBD	2.54	-2.7
	O31	O	GLY177	HBD	2.8	-2.6
	O31	NZ	LYS58	HBA	2.99	-6
ZINC8214398	O19	OD1	ASP111	HBD	2.64	-3.5
	O20	O	LEU30	HBD	2.88	-1.8
	O21	O	MET107	HBD	2.77	-2.5
	O22	O	MET107	HBD	2.65	-2.9
	O24	OD1	ASP178	HBD	2.62	-3.6
	O19	N	ASP111	HBA	2.95	-1.3
	6-ring	CB	ARG161	π-H	4.04	-0.9
ZINC28540146	O18	O	GLU105	HBD	2.6	-4
	O19	O	MET107	HBD	2.64	-3.2
	C21	OD1	ASP111	HBD	3.24	-0.7
	O27	OE2	GLU118	HBD	2.6	-3.5
	O29	O	LEU30	HBD	2.67	-3.1
	O30	OD1	ASP178	HBD	2.71	-3
	O31	O	ARG161	HBD	2.69	-4.4

	O27	N	ASP111	HBA	3.11	-2
	O28	NH1	ARG28	HBA	2.87	-3.9
	6-ring	CB	GLU105	π-H	4.36	-0.6
ZINC4654800	O18	OD1	ASN162	HBD	2.64	-3.3
	O19	OD1	ASP178	HBD	2.6	-2.4
	O30	O	GLY177	HBD	2.64	-2.7
	O32	O	LEU30	HBD	2.72	-2.2
	O10	NZ	LYS58	HBA	2.88	-11.7

Table S3. Physicochemical properties of shortlisted six compounds calculated using the SwissADME server.

compounds	Physicochemical Properties					
	**Molecular weight**	**Num. rotatable bonds**	**Num. H-bond acceptors**	**Num. H-bond donors**	**Consensus Log Po/w (Lipophilicity)**	**Log S (ESOL) Water Solubility**
ZINC3870414	458.37	4	11	8	0.99	-3.56 Soluble
ZINC31155769	418.35	3	10	6	0.35	-3.12 Soluble
ZINC15657732	432.38	3	10	6	0.67	-3.47 Soluble
ZINC8214398	494.49	7	11	6	-0.29	-2.19 Soluble
ZINC28540146	434.35	4	11	7	0.19	-3.27 Soluble
ZINC4654800	448.38	4	11	7	-0.06	-3.18 Soluble

Table S4. Absorption and distribution excretion profile of shortlisted six compounds computed via SwissADME server.

Compounds	Absorption			
	**Synthetic accessibility score**	**P-gp substrate**	**Bioavailability Score**	**BBB**
ZINC3870414	4.20	No	0.56	No
ZINC31155769	5.02	No	0.55	No
ZINC15657732	5.25	Yes	0.55	No
ZINC8214398	6.13	No	0.55	No
ZINC28540146	5.04	No	0.55	No
ZINC4654800	5.29	No	0.55	No

Table S5. Hydrogen bond interaction profile of selected ligands and the reference compound (2XBA) within the ALK active site, based on molecular dynamics simulations. For each interaction, the hydrogen bond acceptor and donor atoms, average bond life (occupancy %), distance (Å), and angle (˚) are listed.

Compounds	Acceptor Atoms	Donor Atoms	Bond Life	Distance (Å)	Angle (˚)
2XBA	GLU105-O	LIG310-N2	73.05%	2.85	153.86
	LIG310-N4	MET107-N	60.77%	2.90	154.94
	ASP178-OD1	LIG310-O34	19.68%	2.70	159.85
	ASP178-OD2	LIG310-O34	18.63%	2.72	159.19
	MET107-O	LIG310-N5	10.49%	2.92	153.63
	LIG310-O34	ALA34-N	5.20%	2.93	159.00
	GLU118-OE1	LIG310-N20	1.49%	2.81	158.51
	GLU118-OE2	LIG310-N20	1.05%	2.83	157.79
	LIG310-O26	LYS58-NZ	0.93%	2.87	146.07
	LIG310-O26	LYS58-NZ	0.85%	2.85	148.23
	LIG310-O26	LYS58-NZ	0.85%	2.86	149.43
	LIG310-O34	LYS58-NZ	0.62%	2.89	154.64
	LIG310-O34	LYS58-NZ	0.52%	2.89	151.57
	LIG310-O34	LYS58-NZ	0.47%	2.89	152.54
	HIE32-O	LIG310-O34	0.11%	2.83	158.63
	GLY177-O	LIG310-O34	0.02%	2.95	155.30
	LIG310-O8	GLY31-N	0.01%	2.94	157.99
ZINC3870414	GLU105-O	LIG310-O11	84.99%	2.77	164.93
	ASP178-OD2	LIG310-O31	57.90%	2.61	162.44
	ASP178-OD1	LIG310-O21	55.21%	2.65	163.38
	GLU75-O	LIG310-O33	53.15%	2.77	147.95
	ASP178-OD2	LIG310-O21	38.65%	2.66	162.64
	ASP178-OD1	LIG310-O31	30.43%	2.61	162.46
	ALA34-O	LIG310-O20	24.00%	2.75	147.59
	GLY36-O	LIG310-O19	23.97%	2.81	149.45
	LIG310-O31	PHE179-N	18.16%	2.92	154.64
	HIE32-O	LIG310-O19	15.80%	2.79	151.12
	GLU75-OE1	LIG310-O32	3.15%	2.67	156.94
	GLU75-OE2	LIG310-O32	2.74%	2.66	160.21
	GLY177-O	LIG310-O31	2.41%	2.72	167.45
	GLU75-O	LIG310-O32	1.58%	2.70	163.85
	HIE32-O	LIG310-O20	1.27%	2.76	150.79
	LIG310-O19	GLY33-N	1.09%	2.94	157.69
	LIG310-O12	MET107-N	0.93%	2.94	147.76
	GLU75-OE1	LIG310-O33	0.61%	2.74	155.26
	ASP111-OD1	LIG310-O21	0.40%	2.68	161.55
	ASP178-OD1	LIG310-O32	0.36%	2.67	160.64
	GLY31-O	LIG310-O19	0.34%	2.79	147.50
	GLU75-OE2	LIG310-O33	0.33%	2.66	161.67

	ASP178-OD1	LIG310-O33	0.12%	2.66	163.02
	ASP178-OD2	LIG310-O32	0.11%	2.69	162.53
	LIG310-O33	LYS58-NZ	0.10%	2.85	153.20
	LIG310-O24	LYS58-NZ	0.07%	2.80	155.86
	LIG310-O21	LYS58-NZ	0.07%	2.93	150.52
	LIG310-O21	ALA34-N	0.07%	2.95	148.74
	LEU30-O	LIG310-O19	0.06%	2.80	154.64
	MET107-O	LIG310-O12	0.05%	2.73	159.60
	PHE179-O	LIG310-O32	0.05%	2.83	142.27
	LIG310-O21	LYS58-NZ	0.05%	2.90	160.52
	LIG310-O31	GLY180-N	0.05%	2.98	156.51
	LIG310-O24	LYS58-NZ	0.05%	2.91	145.04
	LIG310-O21	LYS58-NZ	0.05%	2.91	150.47
	LIG310-O33	PHE179-N	0.04%	2.92	161.05
	LIG310-O19	HIE32-N	0.04%	2.96	152.15
	LIG310-O20	GLY36-N	0.04%	2.97	139.13
	GLU75-OE1	LIG310-O31	0.03%	2.73	163.84
	LIG310-O24	LYS58-NZ	0.03%	2.88	151.17
	LIG310-O31	LYS58-NZ	0.03%	2.89	144.59
	LIG310-O20	ALA34-N	0.02%	2.90	147.86
	LIG310-O32	LYS58-NZ	0.02%	2.92	150.76
	LIG310-O20	GLY33-N	0.02%	2.92	140.27
	LIG310-O33	LYS58-NZ	0.02%	2.93	166.15
	LIG310-O32	ALA34-N	0.02%	2.96	160.06
	ARG161-O	LIG310-O21	0.01%	2.77	158.43
	LIG310-O19	LYS58-NZ	0.01%	2.78	135.81
	LIG310-O19	LYS58-NZ	0.01%	2.81	135.39
	ASP178-OD2	LIG310-O33	0.01%	2.91	148.01
	LIG310-O21	ARG161-NH1	0.01%	2.93	140.73
	LIG310-O21	ARG161-NH2	0.01%	2.98	164.20
	LIG310-O32	PHE179-N	0.01%	2.99	142.55
	LIG310-O32	LYS58-NZ	0.01%	2.99	140.10
ZINC31155769	GLU105-O	LIG310-O6	98.90%	2.67	161.66
	GLU75-OE2	LIG310-O5	86.20%	2.65	165.14
	ASP178-OD2	LIG310-O4	34.98%	2.64	162.41
	LIG310-O6	MET107-N	33.83%	2.90	155.44
	HIE32-O	LIG310-O8	21.86%	2.74	157.74
	HIE32-O	LIG310-O7	19.01%	2.76	160.57
	ASP178-OD1	LIG310-O4	15.05%	2.64	164.88
	GLU75-OE1	LIG310-O5	10.75%	2.64	165.43
	ARG161-O	LIG310-O9	9.31%	2.74	159.77
	LEU30-O	LIG310-O9	8.74%	2.80	151.82
	LEU30-O	LIG310-O8	3.65%	2.77	155.31

	LIG310-O8	HIE32-N	2.82%	2.91	156.43
	ASP111-OD2	LIG310-O9	2.15%	2.68	164.44
	LEU30-O	LIG310-O7	1.23%	2.79	159.57
	ASP111-OD1	LIG310-O9	0.94%	2.72	162.76
	LIG310-O4	ALA34-N	0.77%	2.93	156.27
	ASP111-OD2	LIG310-O8	0.61%	2.80	145.49
	ARG161-O	LIG310-O8	0.35%	2.78	156.81
	ASP178-OD1	LIG310-O5	0.27%	2.72	166.59
	GLY31-O	LIG310-O8	0.25%	2.81	146.65
	LIG310-O5	LYS58-NZ	0.19%	2.90	153.28
	LIG310-O3	ALA34-N	0.13%	2.89	156.04
	ASP111-OD1	LIG310-O8	0.11%	2.80	144.20
	LIG310-O5	PHE179-N	0.11%	2.93	163.59
	LIG310-O5	LYS58-NZ	0.05%	2.92	144.01
	LIG310-O8	GLY33-N	0.05%	2.95	153.88
	LIG310-O7	HIE32-N	0.05%	2.95	145.50
	LIG310-O9	ASP111-N	0.05%	2.96	147.01
	HIE32-ND1	LIG310-O8	0.05%	2.81	153.07
	LIG310-O	ASP111-N	0.05%	2.90	158.59
	LIG310-O7	ALA34-N	0.03%	2.91	163.22
	LIG310-O5	LYS58-NZ	0.03%	2.92	159.67
	LIG310-O3	LYS58-NZ	0.02%	2.84	153.29
	GLY31-O	LIG310-O9	0.02%	2.92	149.70
	LIG310-O4	GLY180-N	0.02%	2.93	136.92
	LIG310-O4	PHE179-N	0.02%	2.95	156.98
	GLY177-O	LIG310-O4	0.01%	2.59	148.00
	LIG310-O3	ARG161-NH1	0.01%	2.80	141.38
	LIG310-O4	LYS58-NZ	0.01%	2.83	162.55
	LIG310-O4	LYS58-NZ	0.01%	2.95	149.10
ZINC15657732	GLU105-O	LIG310-O30	57.36%	2.69	151.69
	ASP178-OD1	LIG310-O26	41.76%	2.67	164.40
	ASP178-OD2	LIG310-O26	23.48%	2.67	164.24
	GLY177-O	LIG310-O29	19.86%	2.79	152.20
	LIG310-O30	MET107-N	15.25%	2.91	154.10
	ARG161-O	LIG310-O25	13.54%	2.75	158.65
	LIG310-O13	MET107-N	12.36%	2.88	153.80
	GLU40-OE1	LIG310-O31	10.95%	2.68	162.46
	ASP111-OD1	LIG310-O25	6.25%	2.70	164.68
	ASP111-OD2	LIG310-O25	4.96%	2.70	164.62
	ALA34-O	LIG310-O27	4.55%	2.80	156.11
	GLU40-OE2	LIG310-O31	4.33%	2.67	162.50
	GLU75-OE2	LIG310-O27	4.30%	2.68	156.06
	LIG310-O27	LYS58-NZ	3.84%	2.87	150.80

	LIG310-O26	ALA34-N	3.80%	2.91	156.95
	LIG310-O27	LYS58-NZ	3.06%	2.87	150.17
	LIG310-O27	LYS58-NZ	2.79%	2.87	149.22
	LIG310-O28	LYS58-NZ	2.13%	2.89	150.26
	LIG310-O27	ALA34-N	2.09%	2.91	154.70
	LIG310-O28	LYS58-NZ	2.03%	2.89	150.96
	LIG310-O28	LYS58-NZ	1.91%	2.90	150.48
	LEU30-O	LIG310-O31	1.78%	2.77	159.53
	ASP178-OD1	LIG310-O27	1.45%	2.75	155.29
	LIG310-O29	LYS58-NZ	1.35%	2.89	151.25
	LIG310-O29	LYS58-NZ	1.32%	2.90	150.21
	LIG310-O29	LYS58-NZ	1.30%	2.90	152.37
	GLY36-O	LIG310-O27	1.14%	2.74	157.63
	ALA34-O	LIG310-O26	1.05%	2.77	159.56
	GLY36-O	LIG310-O28	0.95%	2.82	156.69
	LIG310-O26	LYS58-NZ	0.76%	2.88	149.97
	PHE35-O	LIG310-O27	0.56%	2.78	158.51
	LIG310-O26	LYS58-NZ	0.45%	2.87	150.14
	LIG310-O26	LYS58-NZ	0.32%	2.88	147.69
	SER114-OG	LIG310-O31	0.31%	2.81	162.54
	GLY177-O	LIG310-O28	0.26%	2.76	152.17
	LIG310-O25	HIE32-NE2	0.24%	2.90	157.23
	ASP178-OD2	LIG310-O27	0.23%	2.76	154.58
	GLU75-OE2	LIG310-O26	0.21%	2.72	162.08
	LIG310-O31	ARG28-NH2	0.15%	2.88	149.10
	LIG310-O26	HIE32-NE2	0.13%	2.87	157.17
	LIG310-O31	ARG28-NH2	0.11%	2.90	147.45
	GLY177-O	LIG310-O25	0.10%	2.86	149.70
	HIE32-O	LIG310-O26	0.07%	2.72	156.62
	ASP178-OD2	LIG310-O25	0.06%	2.75	170.66
	LIG310-O27	HIE32-NE2	0.05%	2.86	162.69
	LIG310-O25	ASP111-N	0.05%	2.92	147.69
	ASP178-OD1	LIG310-O25	0.04%	2.66	166.84
	ALA34-N	LIG310-O27	0.02%	2.93	135.67
	HIE32-O	LIG310-O27	0.01%	2.60	152.53
	LIG310-O31	ARG28-NH1	0.01%	2.76	135.88
	PHE35-O	LIG310-O25	0.01%	2.78	141.93
	MET107-O	LIG310-O30	0.01%	2.79	147.09
	HIE32-O	LIG310-O25	0.01%	2.93	142.15
ZINC8214398	ASP111-OD1	LIG310-O23	37.10%	2.72	155.04
	GLU105-O	LIG310-O20	33.59%	2.77	156.96
	GLY177-O	LIG310-O22	28.44%	2.78	154.99
	GLY177-O	LIG310-O21	23.16%	2.76	156.16

	ASP111-OD2	LIG310-O23	8.64%	2.71	157.37
	GLY177-O	LIG310-O23	5.79%	2.76	156.99
	LEU106-O	LIG310-O21	5.49%	2.72	161.42
	MET107-O	LIG310-O21	4.80%	2.75	150.43
	ASP178-OD1	LIG310-O24	3.43%	2.69	157.66
	ASP178-OD2	LIG310-O22	2.90%	2.69	158.75
	ASP178-OD1	LIG310-O22	2.85%	2.69	159.09
	ASP178-OD2	LIG310-O21	2.29%	2.71	164.54
	ARG161-O	LIG310-O23	2.23%	2.79	156.60
	LIG310-O20	MET107-N	2.13%	2.91	162.16
	HIE32-O	LIG310-O24	2.09%	2.74	162.36
	ASP178-OD2	LIG310-O24	2.01%	2.74	155.72
	MET107-O	LIG310-O20	1.83%	2.81	157.25
	LIG310-O22	LYS58-NZ	1.80%	2.89	153.31
	ASP178-OD1	LIG310-O21	1.73%	2.71	163.78
	LIG310-O22	LYS58-NZ	1.72%	2.89	152.15
	LEU106-O	LIG310-O20	1.70%	2.75	158.00
	LIG310-O22	LYS58-NZ	1.53%	2.89	152.42
	GLY177-O	LIG310-O24	1.18%	2.83	156.70
	LEU106-O	LIG310-O22	1.10%	2.75	158.93
	LIG310-O24	LYS58-NZ	0.79%	2.89	151.55
	ASP111-OD1	LIG310-O19	0.62%	2.69	161.66
	GLU105-O	LIG310-O21	0.35%	2.84	160.04
	LIG310-O24	LYS58-NZ	0.34%	2.91	153.39
	ASP178-OD2	LIG310-O23	0.33%	2.73	159.98
	MET107-O	LIG310-O22	0.31%	2.75	160.00
	LIG310-O22	MET107-N	0.18%	2.92	151.57
	ASP178-OD1	LIG310-O23	0.16%	2.76	159.29
	ASP111-OD2	LIG310-O19	0.09%	2.64	163.75
	LIG310-O24	LYS58-NZ	0.09%	2.88	149.22
	LEU30-O	LIG310-O19	0.08%	2.86	146.57
	LIG310-O19	ASP111-N	0.05%	2.94	148.72
	LIG310-O24	ALA34-N	0.05%	2.88	154.97
	LIG310-O23	ASP111-N	0.02%	2.92	143.60
	LEU30-O	LIG310-O20	0.01%	2.69	160.06
	ALA34-O	LIG310-O24	0.01%	2.74	150.29
	LIG310-O20	ARG28-NH2	0.01%	2.92	166.99
	LIG310-O21	MET107-N	0.01%	2.95	155.02
	LIG310-O24	HIE32-NE2	0.01%	2.97	137.91
ZINC28540146	GLU105-O	LIG310-O18	97.40%	2.70	165.28
	ASP178-OD1	LIG310-O31	18.79%	2.67	165.99
	LEU30-O	LIG310-O28	15.37%	2.71	163.89
	ASP178-OD2	LIG310-O31	14.55%	2.69	165.73

	ASP111-OD2	LIG310-O27	13.91%	2.70	160.75
	ASP111-OD1	LIG310-O27	12.37%	2.68	161.25
	HIE32-O	LIG310-O28	10.95%	2.72	160.53
	ASP111-OD2	LIG310-O29	9.66%	2.68	166.45
	LIG310-O28	HIE32-N	8.51%	2.92	159.62
	LIG310-O25	ASP111-N	8.45%	2.91	156.02
	ASP178-OD1	LIG310-O30	6.71%	2.65	163.61
	ASP111-OD1	LIG310-O29	6.59%	2.67	166.37
	GLY36-O	LIG310-O30	6.44%	2.78	144.39
	LIG310-O25	HIE32-N	6.01%	2.89	156.68
	HIE32-O	LIG310-O29	5.85%	2.77	159.18
	GLY33-O	LIG310-O30	5.35%	2.80	149.06
	LIG310-O27	ASP111-N	4.87%	2.91	158.70
	LEU30-O	LIG310-O29	1.99%	2.76	162.39
	HIE32-O	LIG310-O27	1.64%	2.76	159.30
	ASP111-OD2	LIG310-O28	1.07%	2.65	162.17
	LIG310-O19	MET107-N	1.00%	2.93	148.69
	ALA34-O	LIG310-O30	0.70%	2.82	151.33
	LIG310-O27	HIE32-N	0.63%	2.92	157.80
	LEU30-O	LIG310-O27	0.56%	2.75	160.57
	ARG161-O	LIG310-O29	0.45%	2.81	159.17
	HIE32-O	LIG310-O30	0.40%	2.80	145.46
	LIG310-O30 L	LYS58-NZ	0.38%	2.92	151.31
	ARG161-O	LIG310-O28	0.35%	2.73	159.02
	GLY109-O	LIG310-O27	0.35%	2.77	157.52
	MET107-O	LIG310-O19	0.30%	2.72	155.20
	LIG310-O29	ASP111-N	0.30%	2.91	155.37
	ASP111-OD1	LIG310-O28	0.28%	2.66	164.37
	ARG161-O	LIG310-O31	0.19%	2.76	163.11
	LIG310-O30 L	LYS58-NZ	0.16%	2.89	152.21
	GLY33-O	LIG310-O31	0.12%	2.71	157.56
	LIG310-O31	ALA34-N	0.10%	2.94	156.27
	GLU118-OE2	LIG310-O27	0.09%	2.74	156.89
	HIE32-ND1	LIG310-O27	0.08%	2.86	150.83
	LIG310-O31 L	LYS58-NZ	0.08%	2.90	155.49
	GLY36-O	LIG310-O31	0.06%	2.78	159.59
	LIG310-O30 L	LYS58-NZ	0.06%	2.88	141.30
	LIG310-O31 L	LYS58-NZ	0.06%	2.95	148.40
	GLY31-O	LIG310-O29	0.05%	2.84	146.42
	SER114-OG	LIG310-O27	0.05%	2.82	156.73
	LIG310-O30	ALA34-N	0.04%	2.90	156.56
	LIG310-O28	ASP111-N	0.04%	2.92	157.76
	GLY109-O	LIG310-O19	0.03%	2.86	136.47

	LIG310-O30	GLU37-N	0.03%	2.90	149.99
	LIG310-O30	GLY33-N	0.03%	2.92	158.05
	ASP178-OD2	LIG310-O30	0.02%	2.70	157.49
	LIG310-O31 L	LYS58-NZ	0.02%	2.87	142.97
	HIE32-ND1	LIG310-O28	0.01%	2.79	153.24
	SER114-OG	LIG310-O29	0.01%	2.92	144.99
	LIG310-O20	HIE32-N	0.01%	2.96	149.09
ZINC4654800	GLU105-O	LIG310-O32	85.70%	2.70	163.06
	GLU75-OE2	LIG310-O30	36.59%	2.67	163.35
	GLU75-OE2	LIG310-O29	24.37%	2.64	160.60
	GLU75-OE1	LIG310-O30	23.65%	2.67	162.42
	GLU75-OE1	LIG310-O29	17.00%	2.67	156.82
	LIG310-O10	LYS58-NZ	12.88%	2.80	151.78
	LIG310-O10	LYS58-NZ	12.75%	2.80	151.77
	GLY177-O	LIG310-O31	11.97%	2.76	161.26
	LIG310-O10	LYS58-NZ	11.66%	2.81	150.25
	GLU75-OE2	LIG310-O31	11.42%	2.62	162.85
	ASP178-OD2	LIG310-O28	11.35%	2.71	164.08
	ASP178-OD1	LIG310-O28	11.23%	2.71	164.25
	GLY36-O	LIG310-O28	7.82%	2.73	163.08
	ASP178-OD2	LIG310-O29	6.53%	2.70	160.63
	ASP178-OD1	LIG310-O19	6.35%	2.61	167.11
	GLU75-OE1	LIG310-O31	6.29%	2.66	162.62
	GLY177-O	LIG310-O29	5.24%	2.74	151.01
	ASP178-OD1	LIG310-O29	3.54%	2.70	162.43
	ARG161-O	LIG310-O30	3.46%	2.77	159.89
	LIG310-O10	ALA34-N	3.21%	2.88	152.39
	LIG310-O26	LYS58-NZ	2.74%	2.89	148.51
	LIG310-O29	LYS58-NZ	2.42%	2.86	150.58
	LIG310-O26	LYS58-NZ	2.35%	2.88	149.05
	ASP178-OD2	LIG310-O30	2.32%	2.67	162.52
	LIG310-O29	LYS58-NZ	2.03%	2.86	150.35
	ARG161-O	LIG310-O18	2.00%	2.68	155.99
	GLY177-O	LIG310-O30	2.00%	2.75	161.05
	LIG310-O26	LYS58-NZ	2.00%	2.89	147.88
	LIG310-O29	LYS58-NZ	1.93%	2.85	150.95
	ASP178-OD1	LIG310-O30	1.92%	2.69	163.10
	LEU30-O	LIG310-O18	1.15%	2.71	156.66
	ASP111-OD2	LIG310-O18	0.99%	2.64	159.80
	LIG310-O32	MET107-N	0.92%	2.91	158.26
	ASN162-OD1	LIG310-O18	0.91%	2.73	158.08
	GLU75-OE2	LIG310-O28	0.89%	2.65	159.99
	ASP178-OD2	LIG310-O31	0.80%	2.70	165.05

	LIG310-O19	ALA34-N	0.65%	2.91	151.19
	LIG310-O28	LYS58-NZ	0.57%	2.86	150.33
	GLY177-O	LIG310-O28	0.50%	2.77	156.25
	ALA108-O	LIG310-O18	0.47%	2.76	160.91
	LIG310-O28	LYS58-NZ	0.40%	2.85	152.45
	LIG310-O28	LYS58-NZ	0.39%	2.86	148.14
	ALA34-O	LIG310-O28	0.38%	2.80	160.75
	LIG310-O31	LYS58-NZ	0.29%	2.86	145.19
	ASP111-OD1	LIG310-O18	0.26%	2.64	160.39
	ASP178-OD1	LIG310-O31	0.23%	2.77	155.78
	LIG310-O28	ALA34-N	0.23%	2.88	155.93
	LIG310-O31	LYS58-NZ	0.19%	2.88	144.39
	LIG310-O20	LYS58-NZ	0.15%	2.89	157.70
	LEU30-O	LIG310-O32	0.15%	2.75	163.84
	LIG310-O20	LYS58-NZ	0.12%	2.91	155.29
	LIG310-O18	ASP111-N	0.08%	2.95	153.83
	ALA34-O	LIG310-O29	0.05%	2.92	149.78
	ASP178-OD2	LIG310-O19	0.04%	2.68	167.17
	ASP111-OD1	LIG310-O31	0.03%	2.77	146.75
	LIG310-O20	LYS58-NZ	0.03%	2.93	162.46
	LEU30-O	LIG310-O19	0.02%	2.61	167.03
	GLY31-O	LIG310-O18	0.02%	2.76	164.63
	ARG161-O	LIG310-O29	0.02%	2.78	157.87
	HIE32-O	LIG310-O19	0.02%	2.82	143.10
	LIG310-O31	LYS58-NZ	0.02%	2.84	142.65
	ASP111-OD2	LIG310-O31	0.02%	2.91	143.56
	LIG310-O10	HIE32-N	0.02%	2.91	154.68
	LIG310-O19	GLY33-N	0.01%	2.87	137.08
	GLY36-O	LIG310-O19	0.01%	2.89	137.99
	LIG310-O30	LYS58-NZ	0.01%	2.98	141.09
